# Activating Effects of the Bioactive Compounds From Coffee By-Products on FGF21 Signaling Modulate Hepatic Mitochondrial Bioenergetics and Energy Metabolism *in vitro*

**DOI:** 10.3389/fnut.2022.866233

**Published:** 2022-03-22

**Authors:** Miguel Rebollo-Hernanz, Yolanda Aguilera, Maria A. Martín-Cabrejas, Elvira Gonzalez de Mejia

**Affiliations:** ^1^Department of Production and Characterization of Novel Foods, Institute of Food Science Research, CIAL (UAM-CSIC), Madrid, Spain; ^2^Department of Agricultural Chemistry and Food Science, Universidad Autónoma de Madrid, Madrid, Spain; ^3^Department of Food Science and Human Nutrition, University of Illinois at Urbana-Champaign, Urbana, IL, United States

**Keywords:** coffee silverskin, coffee husk, fibroblast growth factor, liver cells, metabolism, mitochondria

## Abstract

Coffee by-products contain bioactive compounds that have been shown to have the capacity to modulate human metabolism. The goal of this study was to investigate the effects of the main bioactive compounds in coffee by-products and two aqueous extracts from the coffee husk and silverskin on the activation of fibroblast growth factor 21 (FGF21) signaling and the subsequent regulation of mitochondrial bioenergetics and lipid and glucose metabolism. HepG2 cells treated with palmitic acid (PA) were used in a non-alcoholic fatty liver disease (NAFLD) cell model. The bioactive compounds from coffee by-products (50 μmol L^−1^) and the aqueous extracts from the coffee silverskin and coffee husk (100 μg mL^−1^) increased ERK1/2 phosphorylation and the secretion of FGF21 (1.3 to 1.9-fold). Coffee by-products' bioactive compounds counteracted inflammation and PA-triggered lipotoxicity. Oxidative stress markers (ROS, mitochondrial superoxide, and NADPH oxidase) and the activity of antioxidant enzymes (superoxide dismutase and catalase) were modulated through the activation of Nrf2 signaling. Mitochondrial bioenergetics were regulated by enhancing respiration and ATP production via PGC-1α, and the expression of oxidative phosphorylation complexes increased. Coffee by-products' bioactive compounds decreased lipid accumulation (23–41%) and fatty acid synthase activity (32–65%) and triggered carnitine palmitoyltransferase-1 activity (1.3 to 1.7-fold) by activating AMPK and SREBP-1c pathways. The GLUT2 expression and glucose uptake were increased (58–111%), followed by a promoted glucokinase activity (55–122%), while glucose production and phosphoenolpyruvate carboxykinase activity were reduced due to IRS-1/Akt1 regulation. The bioactive compounds from coffee by-products, primarily chlorogenic and protocatechuic acids, could regulate hepatic mitochondrial function and lipid and glucose metabolism by activating FGF21 and related signaling cascades.

## Introduction

Coffee by-products such as coffee husk and silverskin are produced in significant amounts during harvesting and roasting processes. Producers usually discharge them, which establishes certain sustainability challenges. The coffee husk and silverskin represent around 29 and 4% of the dry weight of the coffee cherry, respectively; therefore, they can be considered a source of compounds with broad bioactivities (1). They contain substantial quantities of antioxidant compounds, such as caffeine (0.1–3.6%) and phenolic compounds (0.1–1.5%), which may be recovered and used to produce value-added healthy components (2). Hence, green extraction methods have been explored to produce extracts from coffee by-products with the goal of promoting the sustainable extraction of phenolic compounds among others (3). Caffeine and chlorogenic acid, the two major compounds found in coffee by-products, have proven *in vitro* and *in vivo* ability to increase insulin production in β-cells and protect the pancreas from oxidative damage in type 2 diabetes conditions (4). Furthermore, our research group has evidenced the potential of coffee by-products phenolic compounds, primarily chlorogenic acid and kaempferol, to reduce adipogenesis, inflammation, oxidative stress, and insulin resistance in adipocytes (5). Owning to their demonstrated capacity to modulate different metabolic pathways under pathological conditions, the compounds from coffee by-products might also regulate hepatic metabolism and thereby prevent non-alcoholic fatty liver disease (NAFLD). Current estimates place the worldwide prevalence of NAFLD at 25% (6). NAFLD is exacerbated by other chronic diseases, such as obesity and type 2 diabetes (7). Increased glucose and saturated fat consumption promote hepatic *de novo* lipogenesis, adipose tissue and liver inflammation, and insulin resistance. It also raises the risk of type 2 diabetes, including hyperglycemia, promoting hepatic *de novo* lipogenesis (8). Hence, nutrition and appropriate dietary habits have the potential to prevent these metabolic diseases (9).

Fibroblast growth factor 21 (FGF21) is a hormone that plays a key role in controlling cellular metabolic processes (10). FGF21 has become an attractive active agent for preventing and treating chronic metabolic diseases because of its benefits on lipid and glucose metabolism. Despite most studies showing that FGF21 has a beneficial impact on metabolism, serum levels of FGF21 are unexpectedly elevated in obese and diabetic individuals, leading to FGF21 resistance (11). FGF21 production is regulated by the peroxisome proliferator-activated receptor α (PPARα) in the liver and PPAR-γ in the adipose tissue, although FGF21 is considered to be produced mainly by hepatocytes (12). The interaction of FGF21 with a co-receptor, β-klotho (β-KL), is required for the effective binding with the receptor of FGF21 (FGFR1) and the subsequent signaling cascade since FGF21 has a very low affinity for FGFR itself (13). The mitogen-activated protein kinase (MAPK) signaling pathway controls FGF21's metabolic activity. Once FGF21 interacts with FGFR/β-KL, it quickly phosphorylates extracellular signal-regulated kinases (ERK)1/2, which are then translocated to the nucleus, triggering the activation of downstream proteins, therefore regulating diverse metabolic processes (14). FGF21 modulates the phosphoinositide 3-kinase (PI3K)/protein kinase B (Akt) and mammalian target of rapamycin (mTOR) pathways in the liver. Furthermore, FGF21 protects the liver from NAFLD-derived lipotoxicity, which causes mitochondrial dysfunction and thereby the production of reactive oxygen species (ROS), and the activation of inflammatory cascades. FGF21 increases energy expenditure by stimulating fatty acid β-oxidation via AMP-activated protein kinase (AMPK), decreasing liver triglyceride levels, and improving glucose homeostasis and insulin sensitivity (15).

There is scarce evidence of the impact of dietary bioactive compounds on the regulation of hepatic FGF21 release or the stimulation of the FGF21 signaling pathway. Few studies have just demonstrated the effects of long-chain fatty acids (16), curcumin (17), betaine (18), and cyanidin-3-glucoside (19) on the secretion of FGF21. Other investigations have evaluated the effects of extracts from fruits (20, 21) or herbal teas (22, 23) on FGF21 signaling. We recently evidenced the effects of cocoa phenolic compounds, especially protocatechuic acid, epicatechin, and catechin, on FGF21 signaling activation (24). Moreover, no research has been conducted on the effect of bioactive compounds derived from coffee by-products on hepatic function under metabolic syndrome conditions. In this regard, computational tools may serve as *in silico* strategies to investigate the affinity of small molecules with proteins and elucidate their mechanisms of action (25). We hypothesized that the bioactive compounds in coffee silverskin and coffee husk aqueous extracts would trigger FGF21 signaling in liver cells, preventing hepatic metabolic disorders. No research has been done on the effects of these by-products or the compounds that are included within them (caffeine, phenolic acids, and flavonols). We aimed to investigate the effects of the bioactive compounds from coffee by-products and two aqueous extracts from the coffee husk and silverskin on FGF21 signaling activation and the consequent regulation of mitochondrial bioenergetics, and the regulation of energy, lipid, and glucose metabolism in the liver using palmitic acid (PA)-stimulated HepG2 cells as an *in vitro* model of NAFLD and *in silico* tools.

## Materials and Methods

### Materials

Minimum Essential Medium (MEM) and sodium pyruvate were acquired from Corning cellgro (Manassas, VA, USA), while fetal bovine serum, penicillin-streptomycin (100×), and 0.25% trypsin-EDTA were obtained from Gibco Life Technologies (Grand Island, NY, USA). Standards (with a purity higher than 96% in all cases) of the major compounds identified in coffee by-product extracts comprised caffeine (CAF), chlorogenic acid (CGA), caffeic acid (CA), protocatechuic acid (PCA), gallic acid (GA), and kaempferol (KMP) were commercially acquired Sigma-Aldrich (St. Louis, MO, USA) and Extrasynthese (Genay, France). Throughout the manuscript, they would be denominated as “bioactive compounds from coffee by-products”. Recombinant human FGF21 was obtained from R&D Systems (Minneapolis, MN, USA), whereas the FGFR inhibition, PD173074, was purchased from AdoQ Bioscience (Irvine, CA, USA).

### Coffee By-Products Aqueous Extracts Preparation and Bioactive Compound Characterization by UPLC-MS/MS

Fortaleza S.A. (Bilbao, Spain) supplied the coffee silverskin from Colombia, while “Las Morenitas” (Los Potrerillos, Nicaragua) supplied the coffee husk, both from the arabica species (*Coffea arabica* L.). Aqueous extracts, rich in caffeine and phenolic compounds from the coffee silverskin and coffee husk, were prepared using extraction methods published previously (3). After grinding (pilot-scale ball mill) and sieving (500 μm), the coffee silverskin (25 g) was poured into boiling (500 mL) water and mixed for 10 min. Milled coffee husk (10 g) was mixed with boiling water (500 mL) and agitated for 90 min. The aqueous extracts from the coffee silverskin (CSE) and the coffee husk (CHE) were filtered (qualitative filter paper grade 4, pore size 25 μm) and frozen at −80°C for 24 h. Frozen extracts were freeze-dried and then stored in sealed flasks, protected from light and humidity, at −20°C until further usage. The bioactive compounds present in the above extracts were assessed using UPLC-ESI-MS/MS and a previously developed chromatographic method (26). Briefly, after filtering (0.22 μm), an internal standard 4-hydroxybenzoic-2,3,5,6-d4 acid solution (Sigma-Aldrich, St Louis, MO, USA) was added to the samples in a 1:5 (v/v) proportion. The column utilized was a Waters BEH-C18 with 2.1 × 100 mm and 1.7 μm particle sizes. The liquid chromatographic system employed a Waters Acquity UPLC (Milford, MA, USA) with a binary pump, a heated autosampler (10°C), and a heated column compartment (40°C). The LC effluent was injected into an Acquity TQD tandem quadrupole mass spectrometer with a *Z*-spray ESI source. A gradient composed of solvents A (water:acetic acid, 98:2 v/v) and B (acetonitrile:acetic acid, 98:2 v/v) was applied at a flow rate of 0.5 mL min^−1^ as follows: 0 min, 0.1% B; 1.5 min, 0.1% B; 11.17 min, 16.3% B; 11.5 min, 18.4% B; 14 min, 18.4% B; 14.1 min, 99.9% B; 15.5 min, 99.9% B; 15.6 min, 0.1% B; and 18 min, 0.1% B. The injection volume was 2 μL. The MRM mode was used to record the transition of the parent and product ions for each compound. Phenolics were analyzed using negative ionization mode, whereas methylxanthines were detected using the positive ionization mode. The ESI parameters were as follows: capillary voltage, 3 kV; source temperature, 130°C; desolvation temperature, 400°C; desolvation gas (N_2_) flow rate, 750 L h^−1^; cone gas (N_2_) flow rate, 60 L h^−1^. All compounds were quantified utilizing the calibration curves of their respective standard solutions.

### Cell Culture Growing Conditions

HepG2 human hepatocytes from the American Type Culture Collection (ATCC, Manassas, VA) were cultured at 37°C, and 5% CO_2_ in MEM supplemented with 10% fetal bovine serum, 1% penicillin-streptomycin, and 1% sodium pyruvate. The cells were seeded in flasks at a density of 5 × 10^5^ cells cm^−2^.

### Cell Culture Model of NAFLD Prevention

Hepatocytes were cultured with the standard solutions of the main compounds found in coffee by-products (CAF, CGA, CA, PCA, GA, and KMP; 5, 10, 20, 50, or 100 μmol L^−1^), CSE or CHE (10, 20, 50, 100, 200, or 500 μg mL^−1^), or FGF21 (5, 10, 20, or 50 nmol L^−1^) for 24 h in the absence (non-treated cells controls, NT) or presence of PA (500 μmol L^−1^). Supernatants were collected, centrifuged, and stored at −80°C until being used. The treated cells were rinsed twice with ice-cold PBS, and then RIPA Lysis Buffer System (Santa Cruz Biotechnology, CA, USA) was added to lyse the cells. The cell suspension was sonicated, centrifuged at 10,000 × *g* for 10 min at 4°C to eliminate cell debris, boiled for 5 min, and then kept at −80°C until further analysis. The protein concentration in cell lysates was quantified with the DC protein assay (BioRad, Richmond, CA, USA).

### Cell Viability Evaluation

The cell viability of hepatocytes treated as indicated in Section Cell Culture Model of NAFLD Prevention was measured colorimetrically using the CellTiter 96 Aqueous One Solution Proliferation assay (Promega Corporation, Madison, WI, USA).

### Evaluation of the Effect of Coffee By-Products' Bioactive Compounds on FGF21 Signaling Activation

#### *In silico* Molecular Docking of the Interaction of Coffee By-Products' Bioactive Compounds With the FGF21 Receptor

The main bioactive compounds found in coffee by-products (CAF, CGA, CA, PCA, GA, and KMP) were analyzed as potential ligands for the separate subunits of the FGF21 receptor through molecular docking. Their 3D crystal structures (FGFR1: 5A4C; β-KL: 5VAQ) were acquired from the Protein Data Bank website (http://www.rcsb.org/pdb/home/home.do, Research Collaboratory for Structural Bioinformatics, US). The bioactive compounds (CAF, CGA, CA, PCA, GA, and KMP) chemicals structures were gathered from the PubChem Compound database (https://pubchem.ncbi.nlm.nih.gov/pubchem, National Center for Biotechnology Information, NIH, US). AutoDock Tools (Scripps Research, Center for Computational Structural Biology, San Diego, CA, US) was employed to import ligand files in order to apply Gasteiger partial charges and calculate the root of each structure set's rotatable bonds. Similarly, AutoDock Tools served to define the search space dimensions, center point, and flexible torsions in the target proteins. The active sites of co-crystallized antagonists or substrates were selected as the center of the docking space. AutoDock Vina (Scripps Research, Center for Computational Structural Biology) was used to conduct the docking calculations. The coffee by-products' bioactive compounds were docked to the FGF21-active site in FGFR1 (*x*, 90.58; *y*, 0.15; *z*, 14.27) and β-KL (*x*, 54.53; *y*, 35.60; *z*, 52.79). For each ligand, one hundred runs were conducted, and the conformation with the strongest binding affinity (lowest binding energy, BE) was registered. Protein-ligand BE and protein-protein (FGF21) BE for the co-crystallized complexes gathered from PBD were calculated using PRODIGY (27). The Discovery Studio 2017 R2 Client (Dassault Systèmes Biovia Corp, San Diego, CA, US) served to examine the protein-ligand interactions and binding modes.

#### ERK1/2 Phosphorylation Assessment

The phosphorylation of ERK1/2 was assayed in hepatocytes' lysates after the treatments indicated in Section Cell Culture Model of NAFLD Prevention using an ELISA kit as specified by the manufacturer (R&D Systems, Minneapolis, MN, USA). To assess the specificity of coffee by-products' bioactive compounds for increasing the phosphorylation of ERK1/2 via FGFR signaling, PD173074 (50 nmol L^−1^) was utilized as a selective FGFR inhibitor.

#### FGF21 Secretion Quantification

The release of FGF21 in HepG2 cells culture media was quantified using commercial ELISA kits following the manufacturer's specifications (R&D Systems, Minneapolis, MN, USA).

### Determination of Protein Expression and Phosphorylation by Western Blot

The effects of the bioactive compounds from coffee by-products on the expression levels of the following proteins: PPARα (sc-398394), phosphorylated^S40^ (PA5-67520) and total (MAB3925) nuclear factor (erythroid-derived 2)-like 2 (Nrf2), peroxisome proliferator-activated receptor-gamma coactivator 1α (PGC-1α, sc-518025), oxidative phosphorylation (OXPHOS) proteins (ab110413), p-AMPK^T172^/AMPK (sc-33524/sc-74461), sterol regulatory element-binding protein 1c (SREBP-1c, sc-17755), fatty acid synthase (FASN, sc-48357), phosphorylated insulin receptor substrate (IRS)-1^S318^/IRS-1, p-Akt1^S473^/Akt1 (sc-293125/sc-5298), and glucose transporter 2 (GLUT-2, SAB1303865), were analyzed by Western blot. Antibodies were purchased in Santa Biotechnology (Santa Cruz, CA), Thermo Fisher Scientific (Rockford, IL), R&D Systems (Minneapolis, MN), and Abcam (Cambridge, UK). Similar quantities of cell lysate protein (15 μg) were separated by electrophoresis using 4–20% gradient SDS–polyacrylamide (SDS-PAGE) gels. Separated proteins were transferred onto PVDF membranes, which were then blocked with 5% (w/v) non-fat dry milk in Tris-buffered saline + 0.1% Tween 20 for 1 h at room temperature. Membranes were incubated with primary antibodies overnight at 4°C. To determine the level of OXPHOS complexes protein expression, the cell lysates mitochondrial fractions were isolated using a commercial kit (Mitochondria Isolation Kit for Cultured Cells, Thermo ScientificTM, Rockford, IL, USA). An identical quantity of mitochondrial protein (5 μg) was loaded to SDS-PAGE, separated electrophoretically, transferred to membranes, and then incubated with a MitoProfile® total OXPHOS WB antibody cocktail (Abcam, Cambridge, UK). All the membranes were then washed and probed with secondary antibodies (1:5,000, 2 h, RT; GE Healthcare, Buckinghamshire, UK). ECL Prime Western Blotting kit (GE Healthcare, Buckinghamshire, UK) was used to reveal the protein bands, and then pictures were obtained on an ImageQuant 800 System (GE Healthcare, Buckinghamshire, UK). Protein loading controls (β-actin and VDAC1, for whole cell lysate and the mitochondrial fraction, respectively) were used to calculate the relative expression of each protein.

### Assessment of the Effect of the Coffee By-Products' Extracts on Metabolic Related Signaling Pathways Phosphorylation Pattern

Hepatocytes cultured as indicated in Section Cell Culture Model of NAFLD Prevention followed a serum starvation incubation for 30 min and a 10 min insulin (10 ng mL^−1^) stimulation. The phosphorylation pattern of the protein from the different cell lysates was assessed as reported by the manufacturer's directions (AAH-INSR and AAH-AKT, RayBiotech, Peachtree Corners, GA, USA). Arrays signal was visualized on an ImageQuant 800 System (GE Healthcare, Buckinghamshire, UK). The complete list of proteins analyzed, their codes, biological functions, phosphorylation sites, and effects can be found in Supplementary Table 1.

### Bioinformatic Analysis

The resultant significantly up-phosphorylated proteins and subsequent protein-protein interactions were examined using Metascape (https://metascape.org) (28). The protein-protein interacting network diagrams and the enrichment analysis of the up-phosphorylated proteins and their nearest functional and predicted associations were also created.

### Assessment of the Effect of the Bioactive Compounds From Coffee By-Products on Hepatic Lipotoxicity

#### Determination of Lactate Dehydrogenase Release

The LDH activity was quantified in cell culture supernatants using the Pierce LDH cytotoxicity assay kit (Thermo Scientific, USA).

#### Quantification of Cytokines Release

Tumor necrosis factor (TNF)-α, interleukin (IL)-6, and IL-1β levels in cell supernatants were determined using ELISA commercial kits of each cytokine following the manufacturer's guidelines (R&D Technologies, Minneapolis, MN, USA).

#### Evaluation of Nitric Oxide Synthase Activity

NOS activity was measured in cell lysates using a total nitric oxide synthase activity kit according to the manufacturer directions (BioVision, Milpitas, CA, USA).

### Assessment of the Effect of the Bioactive Compounds From Coffee By-Products on Oxidative Stress and Mitochondrial Function

#### Evaluation of ROS and Mitochondrial ${\text{O}}_{2}^{•-}$ Production and the Mitochondrial Membrane Potential (Δ*Ψ*m)

ROS production was measured after the treatments described in Section Cell Culture Model of NAFLD Prevention. Hepatocytes were incubated in MEM for 1 h with 2′,7′dichlorodihydrofluorescein diacetate (DCFDA, 25 μmol L^−1^), then washed with PBS, and fluorescence was assessed (485 nm, excitation, and 535 nm, emission). Mitochondrial ${\text{O}}_{2}^{•-}$ was determined by incubating cells with 50 μmol L^−1^ Mitosox Red (Invitrogen Molecular Probes, Carlsbad, CA, USA) and measuring the fluorescent signal at 510/580 nm (excitation/emission). Similarly, the Δ*Ψ*m was assayed applying the mitochondria-specific fluorescent dye, JC1 (Thermo Fisher, Skokie, IL, USA), according to the manufacturer's directions. JC1 aggregates were observed at 550/590 nm (excitation/emission), whereas JC1 monomers were detected at 485/535 nm (excitation/emission). The ratio aggregates/monomers was calculated for each treatment as the Δ*Ψ*m.

#### Evaluation of Enzymatic Antioxidant Activity

The activity of NADPH oxidase was determined as previously explained after the treatments indicated in Section Cell Culture Model of NAFLD Prevention (29). Hepatocytes were incubated with NADPH (250 μmol L^−1^) for 5 min, and NADPH oxidation was evaluated by the difference in the optical density at 340 nm. Superoxide dismutase (SOD) and catalase activities were evaluated in cell lysates. For the SOD activity, cell lysates were incubated with nitro-blue tetrazolium (60 μmol L^−1^) in a final volume of 250 μL (30). The increase in optical density (570 nm) was recorded for 15 min at 37°C. For catalase activity, cell lysates were mixed with 30 mmol L^−1^ H_2_O_2_ and buffer (100 mmol L^−1^ PBS, pH 6.8) (31). The rise in optical density was recorded at 240 nm, for 5 min, at 37°C.

### Assessment of the Effect of the Bioactive Compounds From Coffee By-Products on Mitochondrial Function

After the treatments indicated in Section Cell Culture Model of NAFLD Prevention, Mitotracker Green was used to quantify mitochondrial mass (Mitotracker Deep Green FM, Invitrogen), recording the fluorescence intensity (excitation at 644 nm and emission at 665 nm). The oxygen consumption rate and citrate synthase (CS) activity were determined using two commercial kits following the manufacturer's guidelines (ab197243; Abcam, Cambridge, UK, and Cayman Chemical Item No. 701040, respectively). The activity of the OXPHOS complex I (CI) was evaluated following as previously explained (32). The NADH oxidation rate was utilized to determine the OXPHOS CI activity in the cell lysates' mitochondrial fraction (see Section Determination of Protein Expression and Phosphorylation by Western Blot). ATP production was quantified in cell lysates using a kit and following the manufacturer's directions (Cayman Chemical, No. 700410).

### Assessment of the Effect of the Bioactive Compounds From Coffee By-Products on Lipid Metabolism

#### Determination of Cellular Lipid and Triglyceride Accumulation

The lipid staining with Oil Red O was performed as previously described (33). Briefly, Oil Red O stock solution was prepared by dissolving 0.35% w/v Oil Red O in isopropanol overnight. Cells were fixed with formalin, and a working Oil Red O solution was added to each well. Oil Red O staining was eluted with 100% isopropanol, and the absorbance was measured at 500 nm. Intracellular triglycerides (TAG) were assessed with a commercial kit (Cayman Chemical Item No. 10010303); briefly, cell lysates were mixed with the enzyme mixture solution, and the absorbance was measured at 540 nm, after a 1 h incubation at 37°C.

#### Evaluation of Lipolysis

The culture media collected from HepG2 cells after the treatments indicated in Section Cell Culture Model of NAFLD Prevention was used for quantifying glycerol using a cell-based assay kit (Cayman Chemical Item No. 10011725). Cell supernatants were mixed with the glycerol assay reagent, incubated for 15 min at room temperature, and the absorbance was measured at 540 nm. Comparably, the cell lysates were employed for evaluating lipase activity with a commercial kit (Cayman Chemical Item No. 700640). Briefly, cell lysates were mixed with arachidonoyl-1-thioglycerol and a fluorometric thiol detector reagent, incubated for 15 min at 37°C while monitoring the fluorescent signal produced (385 nm excitation/ 515 emission).

#### Evaluation of FASN Activity

The activity of FASN was measured as previously explained (34). Cell lysates were combined with EDTA (1 mmol L^−1^), dithiothreitol (1 mmol L^−1^), acetyl-CoA (30 μmol L^−1^), and NADPH (0.15 mmol L^−1^). The baseline response was measured at 340 nm for 3 min to assess background NADPH oxidation. FASN activity evaluation was initiated by the addition of malonyl-CoA (50 mol L^−1^), and the optical density was monitored at 340 nm for 20 min.

#### Evaluation of Carnitine Palmitoyltransferase 1 Activity

The activity of CPT-1 was determined in cell lysates, as previously explained (34). A reaction buffer comprising cell lysates, Tris-HCl (100 mmol L^−1^, pH 7.4), DTNB (0.12 mmol L^−1^), BSA (20 μmol L^−1^), TritonX-100 (0.09%), and palmitoyl-CoA (75 μmol L^−1^) was mixed with adding L-carnitine (75 μmol L^−1^), which started CPT-1 activity, and the optical density was measured at 412 nm after 3 min of incubation at 37°C.

### Assessment of the Effect of the Bioactive From Coffee By-Products on Glucose Metabolism

#### Evaluation of Glucose Uptake

The effects of the bioactive compounds from coffee by-products on glucose uptake were assessed using 2-deoxy-2-[(7-nitro-2,1,3-benzoxadiazol-4-yl)amino]-D-glucose (2-NBDG) absorption as previously explained (34). After the treatments indicated in Section Cell Culture Model of NAFLD Prevention, the cells were cultured in glucose-free MEM with 100 μmol L^−1^ 2-NBDG for 2 h. The fluorescence intensity was recorded (485 nm/535 nm excitation/emission wavelengths, respectively) after rinsing the cells with PBS.

#### Evaluation of Glucokinase Activity

The activity of GK was determined using a previously described procedure (34). Glucose-6 phosphate dehydrogenase (100 U mL^−1^) was mixed with glucose, Tris-HCl buffer (pH 9), MgCl_2_, ATP, and NADP. Upon the addition of the cell lysates, the optical density was recorded at 320 nm for 10 min at 37°C. The activity of hexokinase was adjusted by deducting the activity assessed with low concentration glucose (0.5 mmol L^−1^), which only measures low *Km* hexokinases, from the activity recorded with high concentration glucose (360 mmol L^−1^), which evaluates all hexokinases, including GK).

#### Evaluation of Glucose Production

After the treatments indicated in Section Cell Culture Model of NAFLD Prevention, HepG2 cells were seeded in 24-well plates were changed to a glucose production buffer composed of glucose-free MEM, sodium lactate (20 mmol L^−1^), and sodium pyruvate (2 mmol L^−1^) and incubated for 4 h. Subsequently, produced glucose was quantified in the cell media with an Amplex Red Glucose/Glucose Oxidase Assay Kit (Invitrogen, Carlsbad, CA, USA) following the manufacturer's guidelines.

#### Evaluation of Phosphoenolpyruvate Carboxykinase Activity

PEPCK was estimated as previously stated (34). Cell lysates were combined with 50 mmol L^−1^ Tris–HCl, 2 mmol L^−1^ MnCl_2_, 1.25 mmol L^−1^ inosine diphosphate, 50 mmol L^−1^ of KHCO_3_, 2.5 U mL^−1^ malate dehydrogenase, and 0.15 mmol L^−1^ NADH in a 96-well plate. The reaction was started by the addition of 0.4 mol L^−1^ phosphoenolpyruvate and concluded after incubating for 10 min at 37°C by acidifying with 6 mmol L^−1^ HCl and cooling the tubes on ice. The optical density was finally recorded at 340 nm.

### Statistical Analysis

The results were expressed as the mean ± standard deviation (SD) of at least three independent experiments (*n* = 3). Means were compared using the *T*-test or one-way ANOVA and the *post-hoc* Tukey test. Differences among treatments were recognized as significant at *p* < 0.05. Univariate and bivariate analysis of the results was performed with SPSS 24.0. Multivariate analysis (hierarchical clustering and heatmaps) was computed using XLSTAT2021. Graphs were depicted using GraphPad Prism 8.0.

## Results and Discussion

### Caffeine and Chlorogenic Acid Were the Major Bioactive Compounds of Coffee Silverskin and Coffee Husk

**Figure 1:**
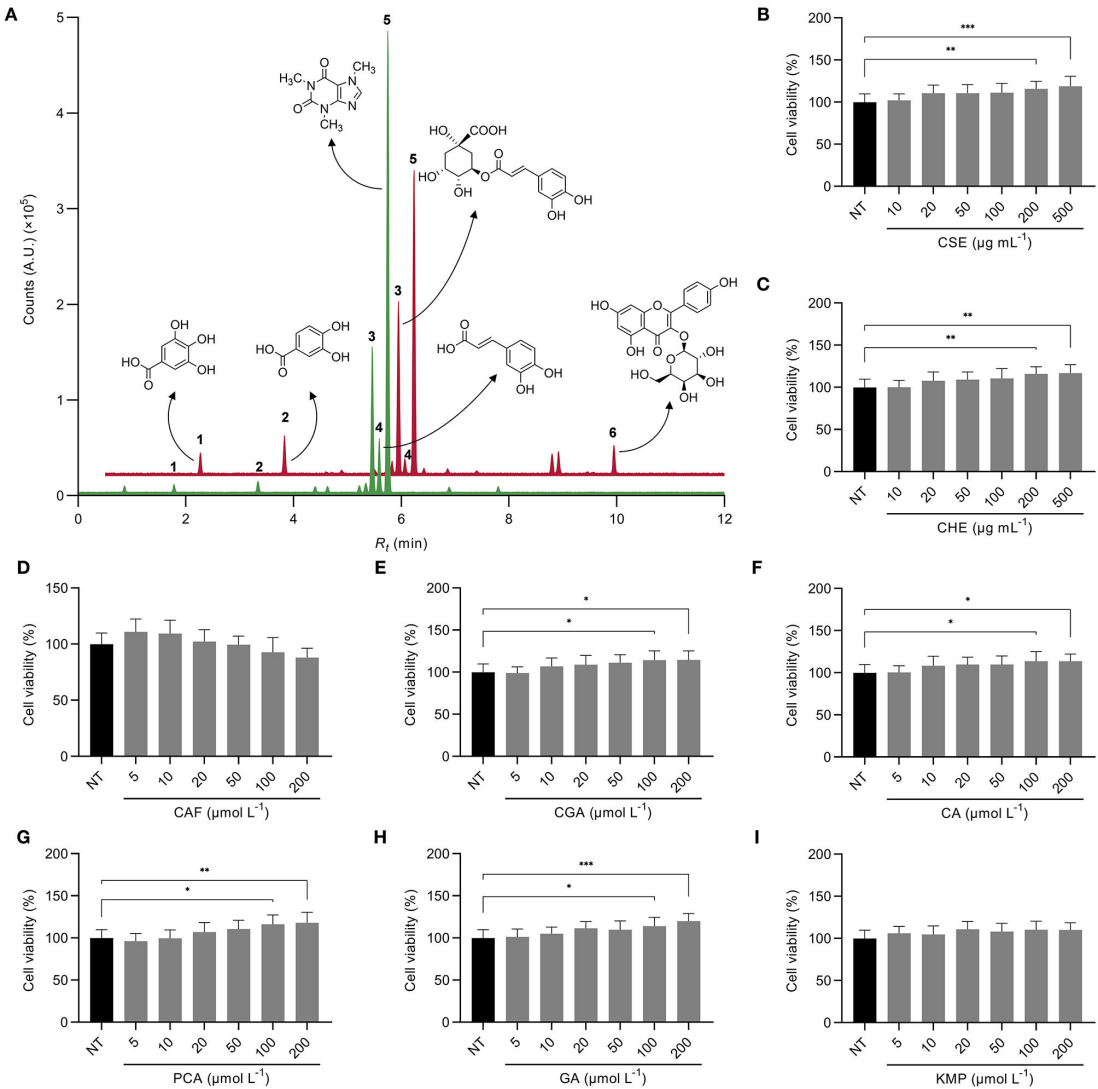
Overlapped UPLC-MS/MS MRM chromatograms of the targeted analysis of bioactive compounds from the coffee silverskin extract (CSE, — red) and the coffee husk extract (CHE, — green) **(A)**. The most abundant compounds were gallic acid (GA, **1**), protocatechuic acid (PCA, **2**), chlorogenic acid (CGA, **3**), caffeic acid (CA, **4**), caffeine (CAF, **5**), and kaempferol-3-*O*-galactoside (KMP, **6**). Cell viability of HepG2 cells treated with CSE **(B)**, CHE **(C)**, CAF **(D)**, CGA **(E)**, CA **(F)**, PCA **(G)**, GA **(H)**, and KMP **(I)**. The results are expressed as mean ± SD (*n* = 3). Asterisks (*, **, or ***) denote significant differences (*p* < 0.05, *p* < 0.01, or *p* < 0.001, respectively) according to the *T-*test between the non-treated control (NT) and each of the concentrations of CSE, CHE, and the standard solutions of the bioactive compounds from coffee by-products.

The UPLC-ESI-MS/MS phytochemical profile of the coffee by-products aqueous extracts showed that chlorogenic (2.8 mg g^−1^, peak 3) and caffeic acids (0.5 mg g^−1^, peak 4) were the main phenolic compound found in CSE, both hydroxycinnamic acids, comprising 97% of the total phenolic compounds measured, but only 15% of the bioactive compounds measured since caffeine (peak 5) was the main compound in this extract (Figure 1A; Supplementary Table 2). Similarly, caffeine was the main bioactive compound found in CHE but at a lower concentration than in the silverskin (9.8 vs. 19.2 mg g^−1^, respectively). In CHE, the main phenolic compound was chlorogenic acid (3.5 mg g^−1^), although protocatechuic acid (peak 2), kaempferol-3-*O*-galactoside (peak 6), and gallic acid (peak 1) were found too, representing 94% of the concentration of studied phenolics (29% of the bioactive compounds measured). Caffeic acid appeared in lower concentrations (57.9 μg g^−1^) in this extract. Previous studies have also investigated the extraction of compounds from coffee by-products using ultrasound-assisted and supercritical extractions and organic or deep eutectic solvents. Similarly, the major phenolic compounds found were chlorogenic, protocatechuic, gallic, and caffeic acids, and the caffeine content was remarkable (35–37). Thus, the primary bioactive compounds present in CSE and CHE were considered as treatments used in the form of standard solutions for the following analyses (kaempferol was tested as aglycone, being the latter the bioavailable form). In basal conditions, none of the treatments, neither CSE, CHE (10–500 μg mL^−1^) nor the standard solutions of the bioactive compounds from coffee by-products (5–200 μmol L^−1^), produced signs of cytotoxicity in HepG2 cells at the analyzed concentrations (*p* > 0.05) (Figures 1B–I). The doses for the following assays were chosen according to the absence of cytotoxicity and their bioactivity reported in earlier studies (CSE and CHE 100 μg mL^−1^, and standard solutions of the bioactive compounds from coffee by-products 50 μmol L^−1^) (24).

### Chlorogenic, Caffeic, and Protocatechuic Acids Emulated FGF21 and Prompted ERK Signaling

**Figure 2:**
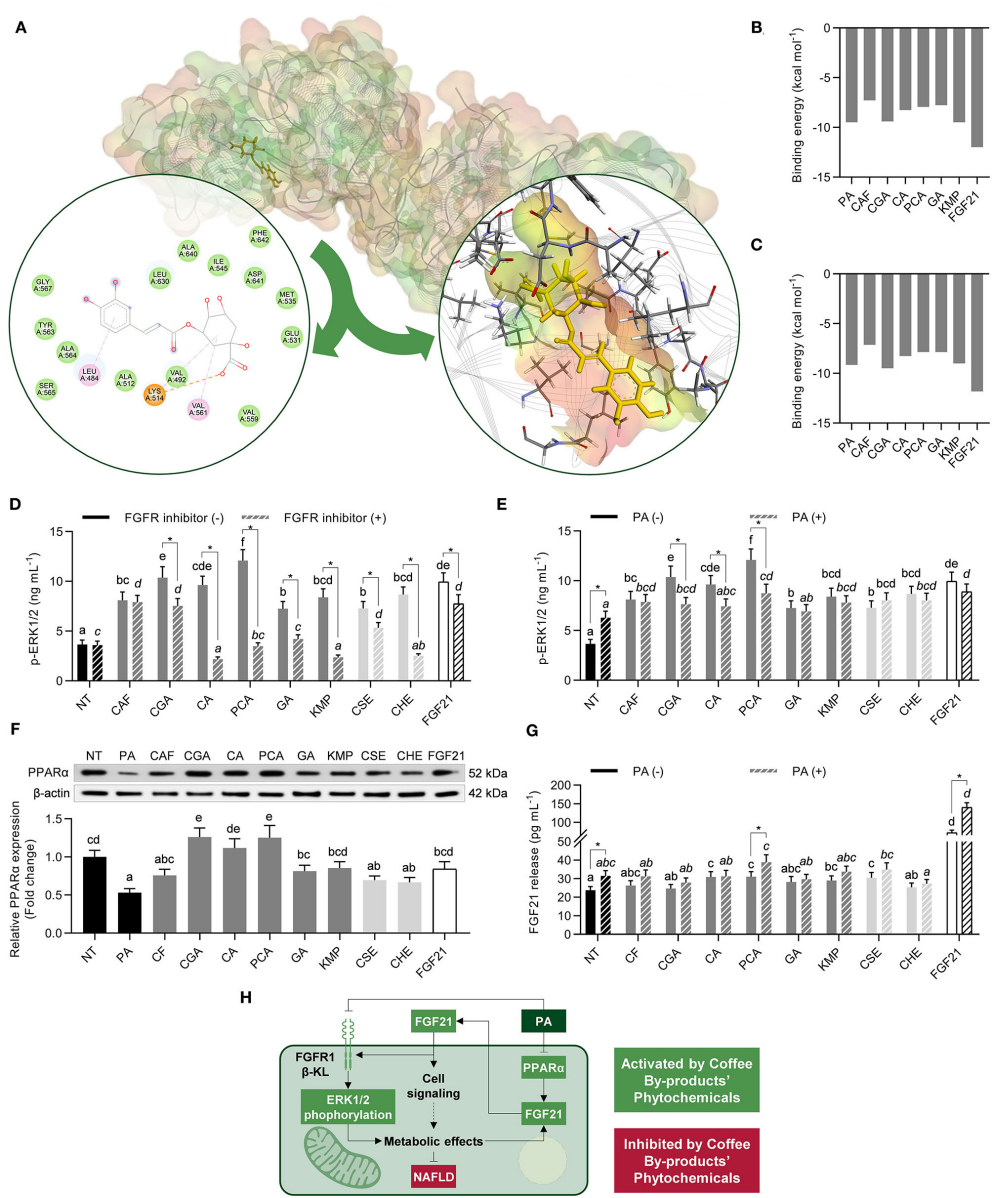
Activating effects of standard solutions of the bioactive compounds from coffee by-products (50 μmol L^−1^), aqueous extracts (CSE and CHE, 100 μg mL^−1^), and FGF21 (20 nmol L^−1^), in the presence of PD173074 (FGFR1 inhibitor, 50 nmol L^−1^) or palmitic acid (PA, 500 μmol L^−1^), on FGF21 signaling in HepG2 human hepatocytes. Coffee by-product's bioactive compounds interacted *in silico* with the subunits of the FGF21 receptor (FGFR) **(A)**, exhibiting different binding energies for FGFR1 **(B)** and β-klotho (β-KL) **(C)**, leading to increases in extracellular signal-regulated kinases (ERK)1/2 phosphorylation **(D,E)**. Simultaneously, HepG2 exhibited increased peroxisome proliferator-activated receptor α (PPARα) protein expression **(F)** and FGF21 release **(G)**. Integrative diagram illustrating the effects of the bioactive compounds from the coffee by-products on FGF21 signaling activation **(H)**. The results are expressed as mean ± SD (*n* = 3). Bars with different letters significantly (*p* < 0.05) differ according to ANOVA and Tukey's multiple range test. Non-italicized letters indicate differences in the absence of FGFR inhibitor or PA, whereas italicized letters indicate differences in the presence of the FGFR inhibitor or PA. Asterisks (*) denote significant differences (*p* < 0.05) according to the *T*-test between two experimental conditions of the same treatment. NT, non-treated cells; CAF, caffeine; CGA, chlorogenic acid; CA, caffeic acid; PCA, protocatechuic acid; GA, gallic acid; KMP, kaempferol; FGF21, fibroblast growth factor 21.

*In silico* molecular docking between the bioactive compounds from coffee by-products, FGF21, or PA and the FRFR1 and β-KL were performed to investigate the possible mechanism of action of the compounds linked with the direct stimulation of the receptor signaling (38). All bioactive compounds demonstrated strong interaction with both FGFR1 and β-KL. The more stable pose for CGA interaction with FGFR1 is shown in Figure 2A. Bioactive compounds' BE with the FGFR1 ranged from −7.3 to −9.5 kcal mol^−1^ (Figure 2B). PA also exhibited strong interactions (−9.5 kcal mol^−1^). In turn, FGF21 demonstrated the lowest binding energy (−12.0 kcal mol^−1^). The tight interaction of the bioactive compounds with the FGF21 activation loop (Ala564, Asp641) via hydrogen bonds, attractive charges, and π-alkyl (strong interactions) was primarily accountable for stabilizing phytochemicals-FGFR1 complexes. Besides, hydrophobic and Van der Waals interactions contributed to the final low BE. Correspondingly, the bioactive compounds from coffee by-products interacted with the β-KL co-receptor exhibiting BE from −7.1 to −9.5 kcal mol^−1^ (Figure 2C). As expected, FGF21's BE was the highest one (−11.8 kcal mol^−1^). Hydrogen bonds with Glu693 also stabilized compounds' interaction with the ligan-binding pocket in the glycoside hydrolase-like domain. This conserved residue interacts with the Ser-Pro-Ser motif of FGF21 C-terminal tail (39), and π-π stacked and π-π T-shaped interactions with Phe826 and Phe931 residues, as well as minor Van der Waals interactions.

Resulting from the interaction with the FGFR1/β-KL complex, HepG2 cells showed increased ERK1/2 phosphorylation (from 2.0 to 3.3-fold, *p* < 0.05) (Figure 2D). CGA, CA, and PCA evoked the highest activating effects, comparable to FGF21. HepG2 cells were co-treated with PD173074, an FGFR1 inhibitor, to confirm that ERK1/2 phosphorylation was FGFR1-dependent. Thus, the phosphorylation of ERK1/2 was reduced by 22–77% (*p* < 0.05), but in the CAF treatment. Given that PA has been shown to disrupt FGF21 signaling (40), p-ERK1/2 levels were measured as well in hepatocytes co-treated with this saturated fatty acid, simulating the microenvironment occurring in the liver during NAFLD conditions (Figure 2E). PA-stimulated cells exhibited a 1.7-fold higher ERK1/2 phosphorylation than NT cells. Nevertheless, only CGA, CA, and PCA treatments exhibited differences in ERK1/2 phosphorylation between PA-treated and NT cells (23–28%, *p* < 0.05). This is the first study investigating the activating effects of the bioactive compounds from coffee by-products' in FGF21 signaling, as far as we know. FGF21-receptor agonists have lately emerged as effective treatments in preventing and managing type 2 diabetes and NAFLD (41). Therefore, we could consider the bioactive compounds from coffee by-products, especially CGA, CA, and PCA, as potential candidate treatments for hepatic metabolism disorders.

Promoting FGF21 release via PPARα activation is another approach to stimulate FGF21 signaling since FGF21 can operate as an autocrine molecule, consequently activating ERK signaling. Although PA reduced PPARα expression, the bioactive compounds from coffee by-products exhibited an activating action by enhancing it, specially CGA, CA, and PCA (1.1 to 2.0-fold, *p* < 0.05) (Figure 2F). In basal conditions, only CA, PCA, KMP, and CSE elevated (22–31%, *p* < 0.05) FGF21 secretion (Figure 2G). In PA-treated hepatocytes, PCA and CSE enhanced FGF21 release by 23 and 11%, respectively (*p* < 0.05). FGF21 interaction with the FGFR1/β-KL complex stimulates the MAPK and then the mTOR/ribosomal protein S6 kinase (S6K) pathways, resulting in an autocrine feedback loop that increases the secretion of FGF21 (Figure 2H) (42). Accordingly, we observed that the release of FGF21 was increased (3.0 and 4.4-fold, *p* < 0.05) in basal conditions and PA-treated hepatocytes, respectively. Even though PA might exert triggering effects on FGF21 secretion, the release of FGF21 was just slightly rose (33%, *p* < 0.05), consistent with prior studies (43). A decrease in FGFR/β-KL expression in response to PA treatment might lead to increased FGF21 resistance. PA exerts adverse effects since it prevents the active signaling of FGF21 (44). Then, we revealed that the primary bioactive compounds from coffee by-products (standard solutions) might activate the ERK pathway via FGFR and, as a result, promote FGF21 release, regardless of the occurrence of PA (Figure 2H). These findings point to the possible role of the bioactive compounds and aqueous extracts from coffee by-products in hepatic cell signaling and downstream metabolic processes.

### Insulin, PI3K-Akt, and mTOR Pathways Governed Hepatocyte Metabolism Changes Under NAFLD Conditions

**Figure 3:**
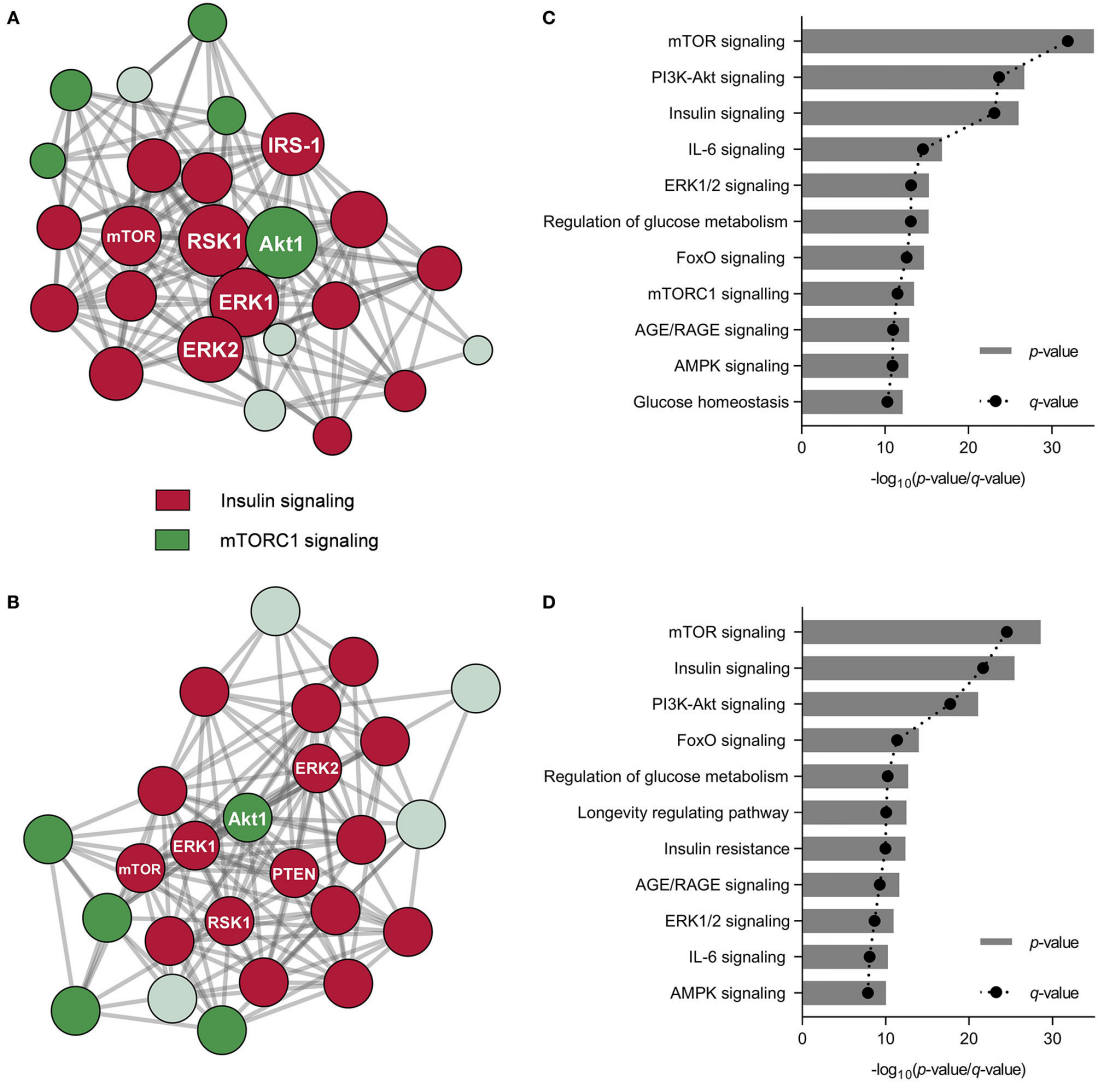
Coffee silverskin (CSE) and coffee husk (CHE) aqueous extracts' bioactive compounds differentially regulated the phosphorylation of proteins associated with cell signaling pathways. Protein-protein interaction networks diagrams from the distinctly phosphorylated protein in HepG2 cells after the co-treatment with palmitic acid (PA, 500 μmol L^−1^) and the aqueous extracts (100 μg mL^−1^) (considering cells just treated with PA as the control group) **(A,B)** and metabolic pathways related to the distinctly phosphorylated proteins **(C,D)**.

As observed, other signaling pathways beyond FGF21/ERK signaling might influence cell metabolism regulation in hepatocytes under NAFLD conditions. The phosphorylation patterns from proteins belonging to key signaling cascades may potentially play a significant role in NAFLD prevention. We investigated the phosphorylation of proteins in PA-challenged HepG2 cells in the presence of CSE and CHE (Table 1). CSE positively regulated the phosphorylation of 26 out of the 27 studied proteins, whereas CHE only 18. It is worth mentioning the phosphorylation changes of the insulin receptor (INSR^Y1189^, 3.3- and 2.4-fold, respectively), IRS-1^S318^ (2.3- and 2.4-fold, respectively), Akt1^S473^ (3.2- and 2.8-fold, respectively), and PTEN^S370^ (5.3- and 4.9-fold, respectively). This phosphorylation pattern results in the activation of insulin/PI3K/Akt signaling pathways (45). Similarly, the increase in AMPKα^T172^ phosphorylation (2.6- and 2.4-fold, respectively) indicates stimulation of energy metabolism-related pathways (46). As previously indicated, CSE and CHE might increase ERK1/2 phosphorylation (3.4- and 1.6-fold, respectively) and activate the mTOR/S6K pathway by phosphorylating mTOR^T2448^ (2.8-fold, only CSE), PRAS40^T246^ (2.7- and 2.5-fold, respectively), and rpS6^S235/6^ (2.3- and 2.5-fold, respectively). Similarly, CSE and CHE induced an increase in the phosphorylation of BAD^S112^ (3.5-fold, both CSE and CHE), GSK3α^S21^ (4.1- and 2.5-fold CSE and CHE, respectively), RSK1^S380^ (2.1- and 2.6-fold, respectively), and RSK2^S368^ (1.9- and 2.6-fold, respectively), notable because it implies a reduction in cell death and an increase in cell proliferation. According to protein-protein interaction and enrichment analysis, most metabolic processes regulated by CSE were also modulated by CHE. Protein-protein interaction diagrams confirmed the associations established among proteins from nearby pathways; CSE and CHE up-phosphorylated proteins were clustered in two groups (insulin and mTORC1 pathways) (Figures 3A,B). The top five processes altered by CSE and CHE, respectively, included insulin, PI3K/Akt, and mTOR signaling (glucose and lipid metabolism), IL-6 signaling (inflammation), and ERK1/2 signaling (Figures 3C,D). Based on the modulation of these multiple signaling pathways, it is expected that biological processes associated to NAFLD such as inflammation, oxidative stress, mitochondrial bioenergetics, and lipid and glucose metabolism will be regulated.

**Table 1:** Effect of coffee by-products' aqueous extracts on the phosphorylation of proteins related to different metabolic pathways in hepatocytes.

**Target protein**	**Phosphosite**	**Effect of phosphorylation**	**Relative phosphorylation**			**Fold change**				**CSE vs. CHE (*p*-value)**
			**PA**	**CSE**	**CHE**	**CSE/PA**	***p*-value**	**CHE/PA**	***p*-value**	
**Insulin signaling**										
IGF1R	Y1165	Induces activity	0.23 ± 0.02	1.03 ± 0.13	0.52 ± 0.05	4.57 ± 0.42	0.008^**^	2.29 ± 0.26	0.025^*^	0.001^**^
INSR	Y1189	Induces activity	0.68 ± 0.05	2.22 ± 0.02	1.61 ± 0.02	3.25 ± 0.14	0.004^**^	2.36 ± 0.13	0.009^**^	0.001^**^
IRS-1	S318	Inhibits molecular association	0.06 ± 0.01	0.14 ± 0.00	0.15 ± 0.00	2.29 ± 0.21	0.030^*^	2.44 ± 0.23	0.026^*^	0.451
SHC-1	Y427	Induces activity	0.18 ± 0.02	0.53 ± 0.09	0.40 ± 0.10	2.93 ± 0.47	0.033^*^	2.24 ± 0.52	0.088	0.163
SHIP-1	Y1020	Induces activity	0.13 ± 0.01	0.42 ± 0.03	0.22 ± 0.03	3.10 ± 0.30	0.015^*^	1.64 ± 0.30	0.130	0.004^**^
SHP-2	T542	Induces molecular association	0.34 ± 0.03	0.88 ± 0.06	0.62 ± 0.06	2.59 ± 0.26	0.019^*^	1.81 ± 0.24	0.063	0.019^*^
**PI3K-Akt-PKB signaling**										
Akt1	S473	Induces activity	0.57 ± 0.04	1.82 ± 0.23	1.61 ± 0.11	3.20 ± 0.36	0.015^*^	2.83 ± 0.24	0.012^*^	0.213
BAD	S112	Inhibits molecular association	0.29 ± 0.02	1.04 ± 0.14	1.04 ± 0.19	3.54 ± 0.36	0.011^*^	3.53 ± 0.45	0.017^*^	0.977
GSK3α	S21	Inhibits activity	0.46 ± 0.03	1.90 ± 0.07	1.13 ± 0.07	4.14 ± 0.20	0.003^**^	2.46 ± 0.19	0.012^*^	0.000^***^
GSK3β	S9	Inhibits activity	0.67 ± 0.02	1.57 ± 0.10	1.90 ± 0.38	2.34 ± 0.15	0.007^**^	2.84 ± 0.39	0.022^*^	0.107
PDK1	S241	Induces activity	0.63 ± 0.02	1.41 ± 0.12	1.56 ± 0.12	2.25 ± 0.18	0.012^*^	2.49 ± 0.18	0.008^**^	0.178
PTEN	S370	Inhibits activity	0.40 ± 0.05	2.09 ± 0.19	1.95 ± 0.16	5.26 ± 0.52	0.009^**^	4.90 ± 0.49	0.010^*^	0.432
**mTOR/S6K signaling**										
4E-BP1	T36	Inhibits activity	0.95 ± 0.03	1.82 ± 0.33	2.70 ± 0.42	1.91 ± 0.29	0.049^*^	2.83 ± 0.32	0.015^*^	0.021^*^
EIF4E	S209	Inhibits molecular interaction	0.10 ± 0.01	0.32 ± 0.04	0.16 ± 0.01	3.11 ± 0.38	0.020^*^	1.56 ± 0.17	0.096	0.003^**^
mTOR	T2448	Induces activity	0.73 ± 0.07	2.05 ± 0.26	1.56 ± 0.23	2.80 ± 0.37	0.025^*^	2.12 ± 0.35	0.057	0.082
p70S6K	T421/S424	Induces activity	0.61 ± 0.02	1.20 ± 0.06	0.85 ± 0.06	1.96 ± 0.12	0.011^*^	1.39 ± 0.13	0.070	0.005^**^
PRAS40	T246	Inhibits activity	0.63 ± 0.04	1.69 ± 0.13	1.56 ± 0.06	2.69 ± 0.21	0.010^*^	2.48 ± 0.14	0.007^**^	0.223
rpS6	S235/236	Induces activity	0.87 ± 0.08	1.97 ± 0.25	2.16 ± 0.27	2.27 ± 0.33	0.041^*^	2.50 ± 0.35	0.032^*^	0.454
**FoxO signaling**										
FOXO3	S413	Induces activity	0.02 ± 0.00	0.06 ± 0.02	0.02 ± 0.00	2.90 ± 0.79	0.083	1.05 ± 0.29	0.860	0.019^*^
p27	T198	Inhibits molecular interaction	0.38 ± 0.05	1.07 ± 0.09	0.95 ± 0.15	2.81 ± 0.37	0.030^*^	2.48 ± 0.45	0.057	0.382
**AMPK signaling**										
AMPKα	T172	Induces activity	0.65 ± 0.03	1.72 ± 0.27	1.57 ± 0.19	2.63 ± 0.33	0.022^*^	2.40 ± 0.26	0.019^*^	0.397
LKB1	S428	Induces activity	0.44 ± 0.05	1.32 ± 0.07	0.77 ± 0.06	2.98 ± 0.30	0.018^*^	1.74 ± 0.26	0.098	0.006^**^
p53	S15	Induces activity	0.60 ± 0.03	1.69 ± 0.13	1.83 ± 0.10	2.83 ± 0.20	0.007^**^	3.07 ± 0.18	0.004^**^	0.197
**MAPK signaling**										
ERK1/2	T202/Y204 Y185/187	Induces activity	0.50 ± 0.02	1.72 ± 0.07	0.78 ± 0.05	3.40 ± 0.16	0.003^**^	1.55 ± 0.15	0.048^*^	0.000^***^
Raf-1	S301	Inhibits activity	0.51 ± 0.06	1.79 ± 0.12	1.44 ± 0.22	3.47 ± 0.34	0.014^*^	2.79 ± 0.45	0.038^*^	0.105
RSK1	S380	Induces protein degradation	0.49 ± 0.03	1.01 ± 0.08	1.26 ± 0.14	2.09 ± 0.20	0.021^*^	2.59 ± 0.27	0.016^*^	0.061
RSK2	S386	Induces activity	0.71 ± 0.03	1.31 ± 0.05	1.88 ± 0.24	1.86 ± 0.10	0.010^**^	2.66 ± 0.27	0.014^*^	0.009^**^

*HepG2 cells were treated with palmitic acid (PA, 500 μmol L^−1^) or co-treated with PA and the aqueous extracts from coffee by-products (CSE and CHE, 100 μg mL^−1^)*.

Results are reported as mean ± SD (*n* = 3). *P*-values followed by *, **, or *** indicate a statistically significance difference between groups when subjected to the *T*-test (*p* < 0.05, *p* < 0.01, *p* < 0.001, respectively).

### Bioactive Compounds From Coffee By-Products, Primarily Chlorogenic Acid and Kaempferol, Prevented Hepatic Lipotoxicity and Inflammation

**Figure 4:**
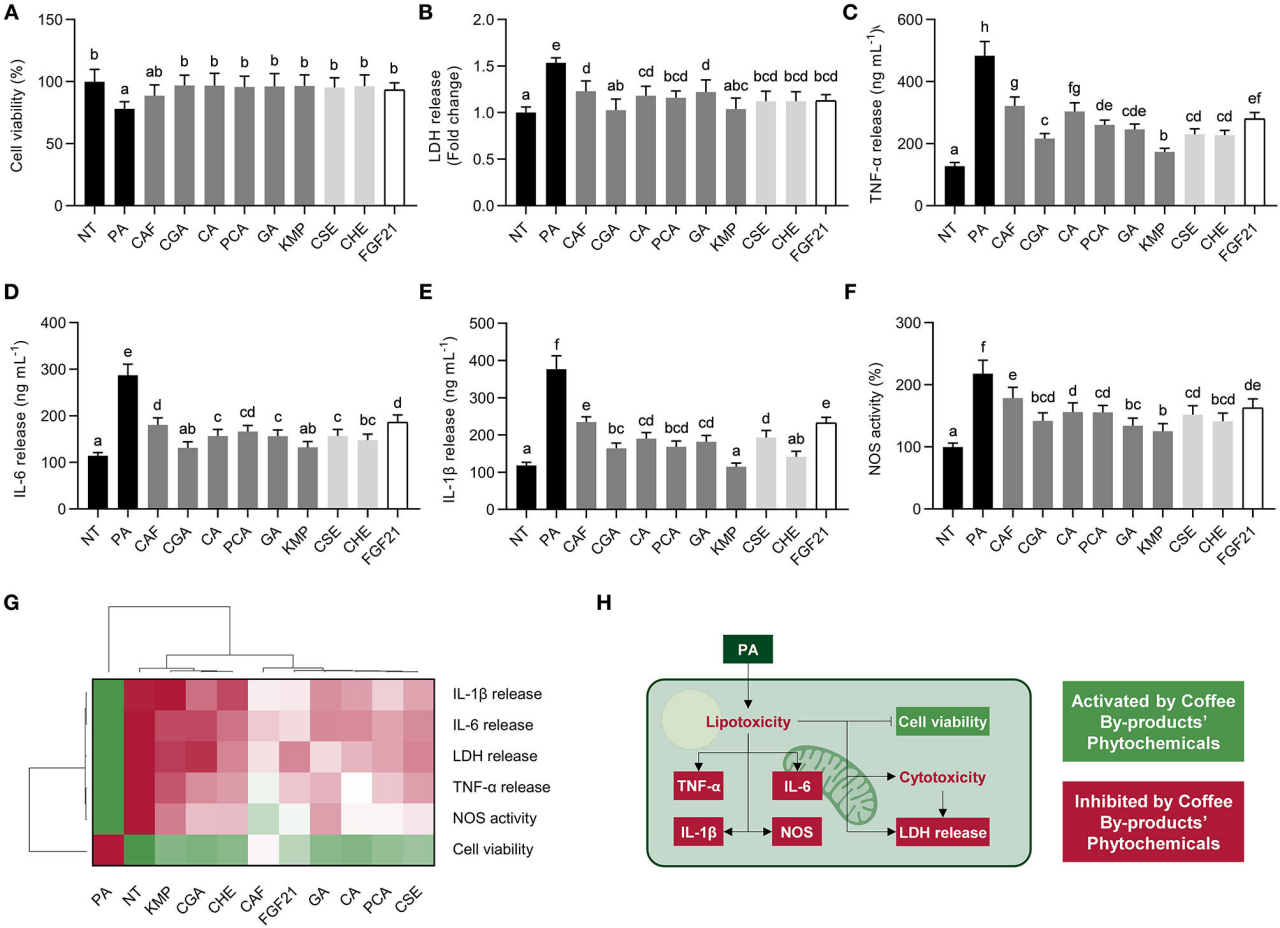
Role of standard solutions of the bioactive compounds from coffee by-products (50 μmol L^−1^), aqueous extracts (CSE and CHE, 100 μg mL^−1^), and FGF21 (20 nmol L^−1^), in the presence of palmitic acid (PA, 500 μmol L^−1^) on lipo/cytotoxicity and inflammation markers in HepG2 human hepatocytes. Coffee by-products' bioactive compounds preserved cell viability **(A)** and diminished lactate dehydrogenase (LDH) release **(B)**. PA-triggered tumor necrosis factor α (TNF-α) **(C)**, interleukin (IL)-6 **(D)**, and IL-1β **(E)** release and nitric oxide synthase (NOS) activity **(F)** were reduced. Hierarchical cluster analysis and heat map [from the lowest (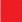 red) to the highest (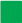 green) value for each parameter] **(G)** and an integrative diagram illustrating the effects of the bioactive compounds from coffee by-products on lipo/cytotoxicity and inflammation **(H)**. The results are expressed as mean ± SD (*n* = 3). Bars with different letters significantly (*p* < 0.05) differ according to ANOVA and Tukey's multiple range test. NT, non-treated cells; CAF, caffeine; CGA, chlorogenic acid; CA, caffeic acid; PCA, protocatechuic acid; GA, gallic acid; KMP, kaempferol; FGF21, fibroblast growth factor 21.

Saturated fatty acids exhibit adverse effects on liver cells (47). We assessed the impact of the standard solutions of the bioactive compounds from coffee by-products and the two aqueous extracts in NAFLD progression in the cell model of PA-stimulated HepG2 cells (Figure 4). Hepatocytes experienced a 22% decrease in cell viability and a 54% rise in LDH release after the PA treatment, showing that HepG2 stimulation with PA induces lipo/cytotoxicity (Figures 4A,B). The standard solutions of the bioactive compounds from coffee by-products, CSE, and CHE, significantly (*p* < 0.05) abolished these effects. Comparably, FGF21 exhibited these protective effects. Concurrently, PA elicited an inflammatory response characterized by the enhanced production of TNF-α, IL-6, and IL-1β (Figures 4C–E). Standard solutions of the bioactive compounds from coffee by-products, CSE, CHE, and FGF21, reduced the secretion of TNF-α by 34–64% (*p* < 0.05). Comparably, IL-6 and IL-1β release were suppressed by 35–54% and 38–70%, respectively. KMP and CGA showed significantly higher effects (*p* < 0.05) in regulating cytokines production than the other compounds and coffee by-products aqueous extracts. PA-treated hepatocytes elicited increased NOS activity (2.2-fold), but the bioactive compounds from coffee by-products counteracted these effects by 18–42% (*p* < 0.05) (Figure 4F). Previous investigations have demonstrated the anti-inflammatory effects of CGA and KMP on LPS-stimulated macrophages, reducing iNOS expression and cytokine release (5). As observed in the hierarchical cluster analysis, all treatments avoided PA's lipotoxic and inflammatory effects (Figure 4G). Nonetheless, CGA, KMP, and CHE exhibited the most effective results, while the phenolic acids, CA, PCA, and GA, demonstrated similar effects to FGF21. Considering that PA can induce cell damage and apoptosis (48), there is a need for dietary and nutraceutical-based treatments for preventing these effects and avoiding hepatic steatosis and the evolution of NAFLD into non-alcoholic steatohepatitis (NASH) (Figure 4H) (49). Besides hepatosteatosis, the inflammatory signaling cascade occurring in the liver cells under the NAFLD microenvironment must be suppressed (50). Previous research has shown that PA activates the nuclear factor kappa-light-chain-enhancer of activated B cells (NF-κB), which dietary phytochemicals can revert (51). The bioactive compounds from coffee by-products proved their capacity to reduce the release of cytokines and the activity of NOS, potentially by downregulating NF-κB and JNK signaling pathways. Furthermore, FGF21 can also decrease cytokine release in PA-stimulated hepatocytes (40). Hence, the bioactive compounds from coffee by-products, primarily CGA and KMP, may slow the progression of NAFLD by reducing the lipotoxic and inflammatory consequences of saturated free fatty acids on the liver (Figure 4H).

### Kaempferol, Among Other Bioactive Compounds From Coffee By-Products, Alleviated Oxidative Stress in Hepatocytes

**Figure 5:**
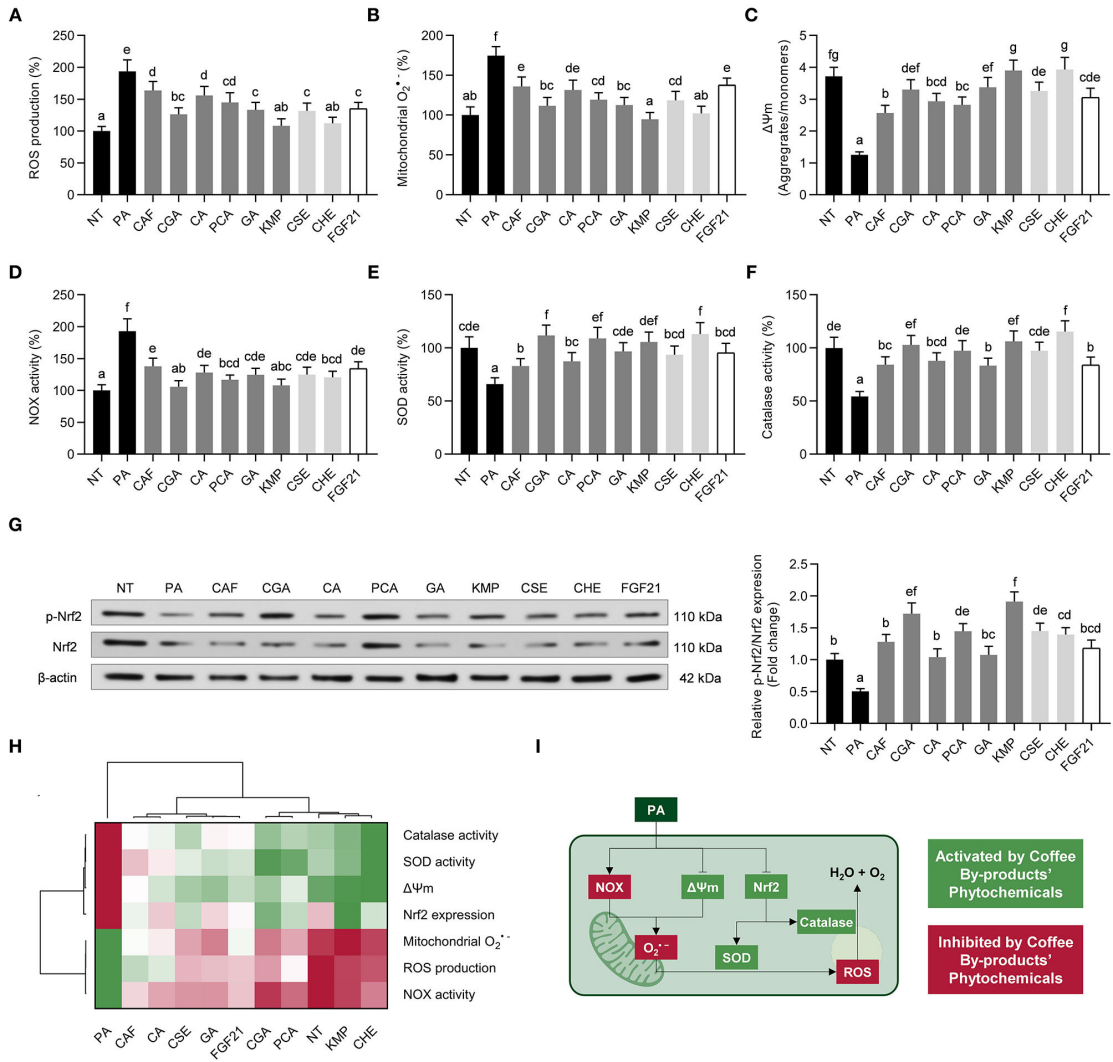
Protective effects of standard solutions of the bioactive compounds from coffee by-products (50 μmol L^−1^), aqueous extracts (CSE and CHE, 100 μg mL^−1^), and FGF21 (20 nmol L^−1^), in the presence of palmitic acid (PA, 500 μmol L^−1^) against oxidative stress in HepG2 human hepatocytes. Coffee by-products' bioactive compounds diminished the production of reactive oxygen species (ROS) **(A)** and mitochondrial ${\text{O}}_{2}^{•-}$ **(B)**, preserving the mitochondrial membrane potential (Δ*Ψ*m) **(C)** in PA-challenged HepG2 cells. The enzymatic activity NADPH oxidase (NOX) **(D)**, superoxide dismutase (SOD) **(E)**, and catalase **(F)** activities, and nuclear factor (erythroid-derived 2)-like 2 (Nrf2) phosphorylation **(G)** were regulated, thereby diminishing oxidative stress. Hierarchical cluster analysis and heat map [from the lowest (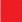 red) to the highest (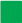 green) value for each parameter] **(H)** and an integrative diagram illustrating the effects of the bioactive compounds from coffee by-products on oxidative stress **(I)**. The results are expressed as mean ± SD (*n* = 3). Bars with different letters significantly (*p* < 0.05) differ according to ANOVA and Tukey's multiple range test. NT, non-treated cells; CAF, caffeine; CGA, chlorogenic acid; CA, caffeic acid; PCA, protocatechuic acid; GA, gallic acid; KMP, kaempferol; FGF21, fibroblast growth factor 21.

Hepatic oxidative stress is another of the consequences of NAFLD. When HepG2 cells are exposed to PA, the presence of oxidative stress markers may indicate cell damage (52). Oxidative stress can be generated in hepatocytes through PA-elicited increased NADPH oxidase activity (NOX) or mitochondrial dysfunction (53). We observed an intensified ROS and mitochondrial ${\text{O}}_{2}^{•-}$ production (1.9- and 1.7-fold, respectively, *p* < 0.05) (Figures 5A,B). Coffee by-products bioactive compounds and FGF21 scavenged ROS (15–44%) and ${\text{O}}_{2}^{•-}$ (21–46%). Likewise, these effects were inversely proportional to the mitochondrial membrane potential, which was decreased by 67% and significantly (*p* < 0.05) enhanced by the bioactive compounds and extracts from coffee by-products and FGF21 (53–109%) (Figure 5C). KMP and CSE fully recovered it. We previously observed the antioxidant activity of kaempferol in both macrophages and adipocytes (5). Concomitantly, NADPH oxidase activity (NOX) was increased by PA (1.9-fold) and decreased by coffee by-products bioactive compounds and FGF21 (29–45%) (Figure 5D). Finally, the bioactive compounds from coffee by-products, both standard solutions and aqueous extracts, promoted the activity of cellular antioxidant defenses. Coffee by-products extracts, standard solution of coffee bioactive compounds, and FGF21 increased the activity of SOD (1.3 to 1.7-fold) and catalase (1.5 to 2.0-fold), which had been reduced by the presence of PA (34 and 46%, respectively) (Figures 5E,F). Coffee pulp extracts and CGA previously demonstrated their ability to reduce H_2_O_2_-triggered oxidative stress and enhance SOD and catalase activities (54). According to some research, NAFLD oxidative stress can be prevented by consuming certain phenolic compounds and vitamin E (52). When exposed to PA, hepatocytes exhibit a less functional phenotype because cell metabolic signals are disrupted. The activation of the ERK and Akt pathways is connected with the stimulation of Nrf2-mediated antioxidative cellular defenses (55). In this regard, the bioactive compounds from coffee by-products protected hepatocytes from the oxidative action of PA by stimulating the Nrf2 pathway (Figure 5G). Coffee bioactive compounds, CGA, PCA, and KMP, to a greater extent, enhanced Nrf2 phosphorylation (2.3 to 3.8-fold, *p* < 0.05), thereby activating a cascade of antioxidant defenses. These compounds have previously demonstrated their ability to regulate Nrf2 antioxidant signaling when used as co-treatment with other oxidative agents (dexamethasone, H_2_O_2_, oleic acid) (56–58). According to the multivariate analysis, the bioactive compounds from coffee by-products effectively reduced PA-induced oxidative stress (Figure 5H). PA stimulation may mimic liver NAFLD conditions by activating NADPH oxidase (NOX) and causing mitochondrial dysfunction in hepatocytes, which undergo oxidative stress (Figure 5I) (52). We found that treatments with the bioactive compounds from coffee by-products reduced both ROS and mitochondrial ${\text{O}}_{2}^{•-}$ generation and the activity of NADPH oxidase. These compounds might be functioning by ROS excess scavenging or boosting the action of SOD and catalase. FGF21 has been found to increase the activity of these antioxidant enzymes in response to an inflammatory environment, resulting in the subsequent conversion of ${\text{O}}_{2}^{•-}$ into H_2_O_2_ and the formation of water and oxygen from H_2_O_2_ (59). Hence, evidence suggests that the bioactive compounds from coffee by-products are working to offset the detrimental effects of PA by activating antioxidant enzymes (SOD, catalase) and maintaining healthy mitochondrial function (see Section Coffee By-Products Bioactive Compounds, Mainly Chlorogenic and Protocatechuic Acids, Promoted Mitochondrial Bioenergetic Functions). Consequently, the studied standard bioactive compounds and aqueous extracts from coffee by-products and FGF21 should be regarded as effective bioactive molecules for mitigating the detrimental effects of free fatty acids causing liver oxidative stress.

### Coffee By-Products Bioactive Compounds, Mainly Chlorogenic and Protocatechuic Acids, Promoted Mitochondrial Bioenergetic Functions

**Figure 6:**
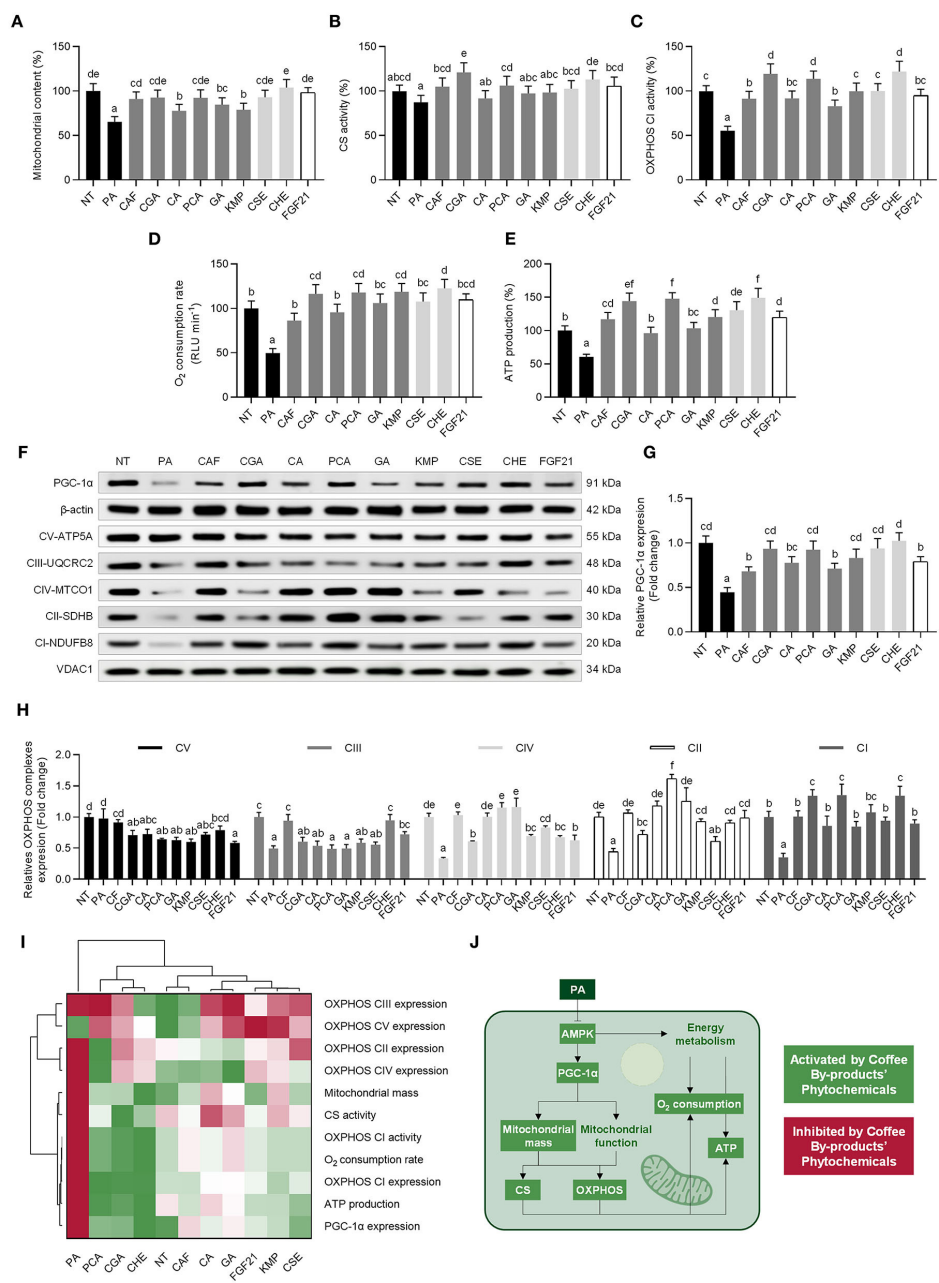
Regulative role of standard solutions of the bioactive compounds from coffee by-products (50 μmol L^−1^), aqueous extracts (CSE and CHE, 100 μg mL^−1^), and FGF21 (20 nmol L^−1^), in the presence of palmitic acid (500 μmol L^−1^) on the mitochondrial bioenergetics of HepG2 human hepatocytes. Coffee by-products' bioactive compounds attenuated the loss of mitochondrial mass **(A)** and mitochondrial function as measured by the citrate synthase (CS) activity **(B)**, oxidative phosphorylation (OXPHOS) complex I (CI) activity **(C)**, O_2_ consumption rate **(D)**, and ATP production **(E)**. The protein expression of the peroxisome proliferator-activated receptor-gamma coactivator 1α (PGC-1α) **(F,G)** and OXPHOS complexes **(H)** was also regulated. Hierarchical cluster analysis and heat map [from the lowest (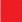 red) to the highest (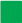 green) value for each parameter] **(I)** and an integrative diagram illustrating the effects of the bioactive compounds from coffee by-products on mitochondrial function **(J)**. The results are expressed as mean ± SD (*n* = 3). Bars with different letters significantly (*p* < 0.05) differ according to ANOVA and Tukey's multiple range test. NT, non-treated cells; PA, palmitic acid; CAF, caffeine; CGA, chlorogenic acid; CA, caffeic acid; PCA, protocatechuic acid; GA, gallic acid; KMP, kaempferol; FGF21, fibroblast growth factor 21.

As stated before, the dysregulation of mitochondrial bioenergetics by PA causes oxidative stress in hepatocytes. An oxidative loop is established when mitochondrial dysfunction leads to an increase in oxidative stress and inflammation (60). Hepatocytes exposed to PA had a 35% reduction in mitochondrial mass, which was reversed by CSE, CHE, the standard solutions of the bioactive compounds in coffee by-products, and FGF21 (36–111%, *p* < 0.05) (Figure 6A). In line with earlier findings, PA stimulation lowered CS activity modestly (13%, *p* < 0.05); however, the bioactive compounds from the coffee by-products completely reversed this effect (Figure 6B) (47). By contrast, the PA challenge harmed the OXPHOS CI activity (45%, *p* < 0.05) (Figure 6C). A significant increase in OXPHOS CI activity was seen from all coffee by-products bioactive compounds and FGF21, even though the effect of CGA, PCA, and CHE was more noteworthy since they increased the activity 2.2, 2.1, and 2.2-fold, respectively (*p* < 0.05). All bioactive compounds from coffee by-products and FGF21 inhibited PA-derived oxygen consumption rate reduction (50%), enhancing it from 1.8 to 2.5-fold (*p* < 0.05) (Figure 6D). ATP is produced through mitochondrial oxidative phosphorylation. Here, we found a 39% decrease in ATP generation, which was mitigated by the bioactive compounds from coffee by-products, which increased it by 1.7 to 2.5-fold (*p* < 0.05) (Figure 6E). Recent research established the role of astaxanthin in alleviating hepatotoxicity and the imbalance in mitochondrial bioenergetics caused by NAFLD *in vitro* and *in vivo* by activating the FGF21/PGC-1α pathways (61). They established that the carotenoid-preventive effects needed FGF21 expression by silencing FGF21 expression. Similarly, the treatment with FGF21 protects mice from developing mitochondrial dysfunction due to NAFLD (62). Here we observed a reduction inf PGC-1α protein expression following PA-stimulation, which was prevented by the bioactive compounds from coffee by-products (30–105%, *p* < 0.05) (Figures 6F,G). Concurrently, the expression of OXPHOS complexes was regulated (Figure 6H). PA-challenge reduced the expression of CI–IV (50–67%, *p* < 0.05). CAF preserved OXPHOS complexes I to IV protein expression. PCA exhibited the most significant effects, enhancing the expression of complexes CIV, CII, and CI (from 3.5 to 3.8-fold, *p* < 0.05). The hierarchical cluster analysis indicated that CGA, PCA, and CHE prevented mitochondrial dysfunction and promoted mitochondrial bioenergetics. The following treatments mimicked FGF21 effects and reversed PA-derived negative impact on hepatocytes (Figure 6I). Dysfunction of the mitochondria accompanies the development of NAFLD (63). OXPHOS activity, O_2_ consumption, and ATP synthesis decrease in this pathological state (Figure 6J). Similarly, other mitochondrial bioenergetic components (citric acid cycle and β-oxidation) are also altered (see Section Phenolic Compounds From Coffee By-Products, Mainly Chlorogenic and Protocatechuic Acids, Reduced *de novo* Lipogenesis and Prompted Fatty Acid Oxidation). Results showed that the bioactive compounds from coffee by-products, primarily CGA and PCA, may help protect the mitochondrial mass, the activity of OXPHOS CI, oxygen consumption rate, and ATP generation in PA-stimulated HepG2 cells. CGA increases ATP synthesis and modulates energy metabolism by increasing AMPK and PGC-1α expression (64). Moreover, CGA protects mitochondrial function and reduces ROS by activating the SIRT-1 pathway (65). In turn, the activation of the AMPK pathway is partially responsible for boosting mitochondrial function 3T3-L1 adipocytes treated with PCA (66). Hepatocyte mitochondrial bioenergetics has also been related to the ERK and Akt pathways (67). Thus, both CGA and PCA seem to be the main bioactive compounds responsible for CHE's potential to prevent mitochondrial dysfunction. Thus, bioactive compounds extracted from coffee by-products should be regarded as active agents in minimizing the development of NAFLD by countering mitochondrial bioenergetics imbalance.

### Phenolic Compounds From Coffee By-Products, Mainly Chlorogenic and Protocatechuic Acids, Reduced *de novo* Lipogenesis and Prompted Fatty Acid Oxidation

**Figure 7:**
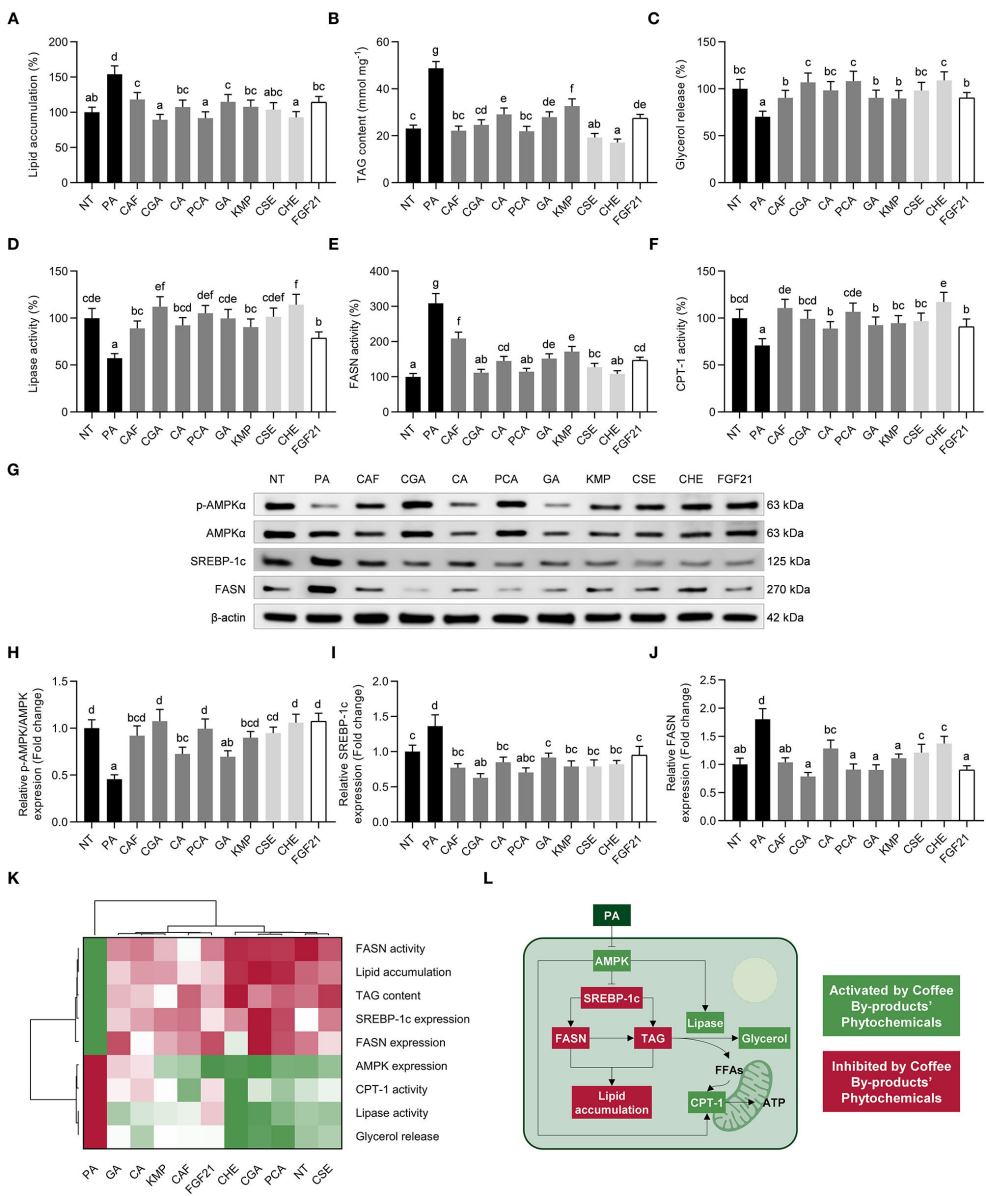
Modulatory effects of standard solutions of the bioactive compounds from coffee by-products (50 μmol L^−1^), aqueous extracts (CSE and CHE, 100 μg mL^−1^), and FGF21 (20 nmol L^−1^), in the presence of palmitic acid (500 μmol L^−1^) on lipid metabolism in HepG2 human hepatocytes. Palmitic acid-treated hepatocytes showed reduced lipid accumulation **(A)**, diminished intracellular TAG content **(B)**, and increased lipolysis measured by glycerol release **(C)** and lipases activity **(D)**. The enzymatic activity of fatty acid synthase (FASN) **(E)** and carnitine palmitoyltransferase 1 (CPT-1) **(F)** and the phosphorylation and expression **(G)** of AMP-activated protein kinase (AMPK)α **(H)**, sterol regulatory element-binding protein (SREBP)-1c **(I)**, and FASN **(J)**, were regulated by bioactive compounds from coffee by-products. Hierarchical cluster analysis and heat map [from the lowest (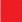 red) to the highest (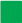 green) value for each parameter] **(K)** and an integrative diagram illustrating the effects of bioactive compounds from coffee by-products on lipid metabolism **(L)**. The results are expressed as mean ± SD (*n* = 3). Bars with different letters significantly (*p* < 0.05) differ according to ANOVA and Tukey's multiple range test. NT, non-treated cells; PA, palmitic acid; CAF, caffeine; CGA, chlorogenic acid; CA, caffeic acid; PCA, protocatechuic acid; GA, gallic acid; KMP, kaempferol; FGF21, fibroblast growth factor 21; FFAs, free fatty acids.

NAFLD is characterized by an accumulation of lipids in the liver (68). PA-challenged hepatocytes showed increased lipid accumulation (1.5-fold, *p* < 0.05) compared to untreated hepatocytes (Figure 7A). The neutral lipid content was considerably decreased by all treatments (53–115%, *p* < 0.05). All treatments significantly decreased lipid accumulation (23–42%, *p* < 0.05). Similarly, the TAG content was reduced by 33–61% (*p* < 0.05) (Figure 7B). The bioactive compounds from coffee by-products reversed the reduced glycerol release (65–130%) and lipase activity (50–134%), indicating an enhancement in the hepatic lipolytic rates (Figures 7C,D). Apart from evaluating the mobilization of free fatty acids, we investigated *de novo* synthesis of fatty acid and β-oxidation by measuring the activity of important enzymes involved in these pathways (FASN and CPT-1) (Figures 7E,F). Coffee by-products' aqueous extracts and the standard solutions of their bioactive compounds inhibited (32–65%, *p* < 0.05) the increased FASN activity, indicating an attenuation of hepatic *de novo* fatty acid synthesis. Furthermore, CPT-1 activity was induced by the standard solutions of the compounds found in coffee by-products, CSE, CHE, and FGF21, counteracting PA-derived effects (62–159%, *p* < 0.05). As previously documented, FGF21 inhibits the production of FASN and increases the activity of CPT-1, therefore decreasing lipid storage and promoting fatty acid oxidation (40). CGA diminished fat accumulation in PA-treated HepG2 cells by inducing PPARα and reducing CD36 expression (54). PCA has been shown to regulate lipid metabolism by decreasing FASN expression and activity and promoting the expression of CPT-1 via the mitochondrial deacetylase sirtuin 3 (SIRT3) pathway in the liver of high-fat-fed mice (69). Activating the AMPK pathway reduces lipogenesis and enhances β-oxidation in the liver. The inhibition of CPT-1, the mitochondrial fatty acid oxidation rate-limiting enzyme, occurs when AMPK inactivates the acetyl-CoA carboxylase (ACC) (70). In this regard, we observed enhanced AMPKα^T172^ phosphorylation (Figures 7G,H). The bioactive compounds from coffee by-products, mainly CAF, CGA, and PCA, stimulated the phosphorylation of AMPK (1.5 to 2.4-fold) and, consequently, reduced the expression of SREBP-1c (46–70%, *p* < 0.05) (Figure 7I) and FASN (44–76%, *p* < 0.05) (Figure 7J). Multivariate analysis indicated that CSE, CHE, CGA, and PCA were the main treatments that regulated lipid metabolism and dampened the detrimental effects of PA in hepatocytes (Figure 7K). Consistently, we have established that the main bioactive compounds from coffee by-products control these pathways (Figure 7L). Accordingly, FGF21 pathway signaling is activated due to FGF21's effects on the ERK, Akt, mTOR, and AMPK pathways (15). Thus, the bioactive compounds from coffee by-products, specifically CGA and PCA, might imitate the activity of FGF21, thereby protecting liver cells from the accumulation of free fatty acids. CGA has evidenced its ability to reduce lipid accumulation in rat livers via AMPK and CPT-1 activation (71). Correspondingly, previous reports have demonstrated that PCA mimics insulin and alters downstream signaling pathways, including the AMPK pathway (72). Thus, the bioactive compounds found in coffee by-products aqueous extracts, CSE and CHE, notably CGA and PCA, can modulate critical hepatic fat accumulation routes, avoiding NAFLD development.

### Coffee Husk Phenolics, Chlorogenic and Protocatechuic Acids, Mimicked FGF21 Regulating Glucose Homeostasis by Promoting Glucose Uptake and Diminishing Gluconeogenesis

**Figure 8:**
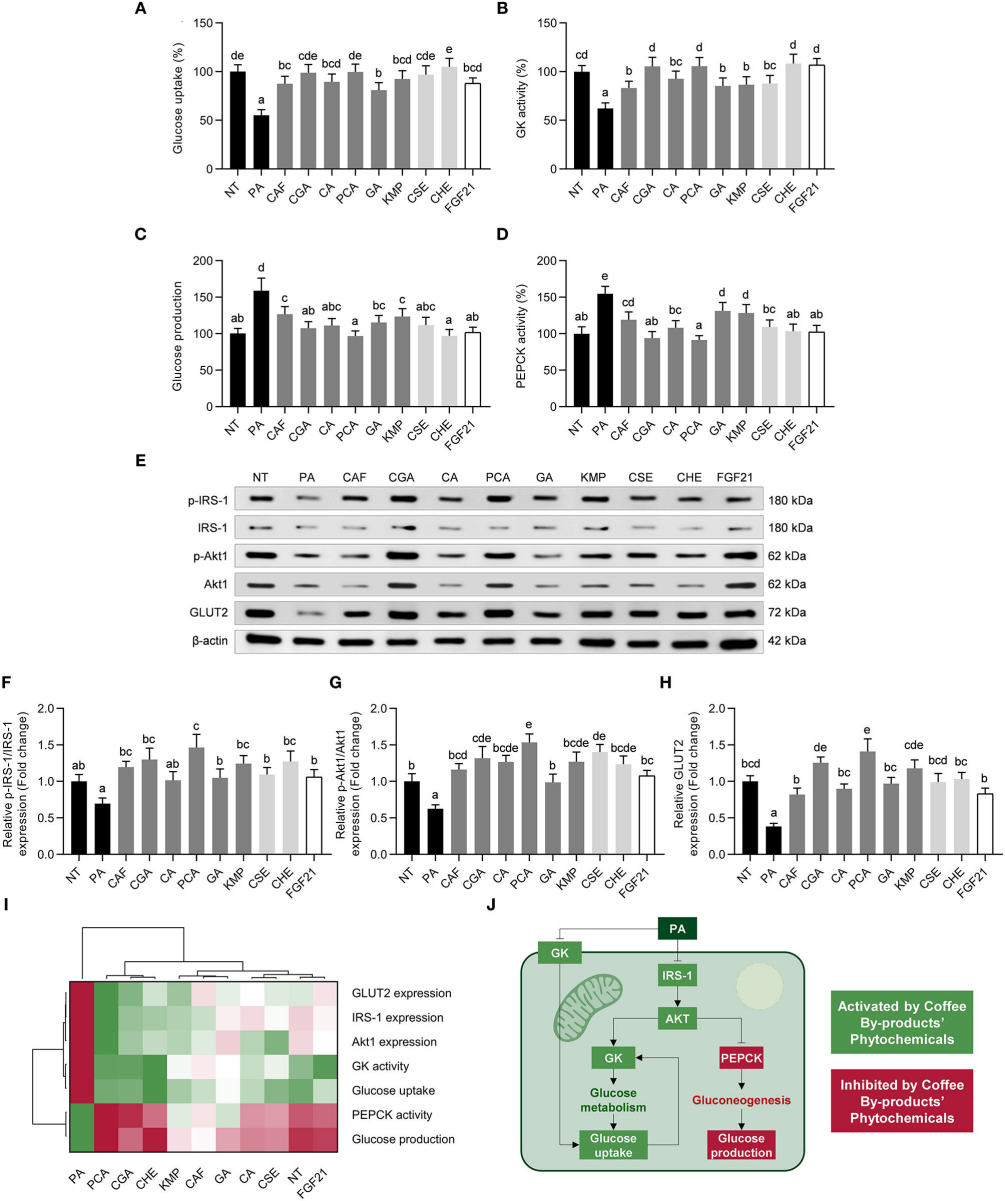
Regulatory role of standard solutions of the bioactive compounds from coffee by-products (50 μmol L^−1^), aqueous extracts (CSE and CHE, 100 μg mL^−1^), and FGF21 (20 nmol L^−1^), in the presence of palmitic acid (500 μmol L^−1^) on glucose metabolism in HepG2 human hepatocytes. Hepatocytes exhibited a modulation on glucose uptake **(A)**, glucokinase (GK) activity **(B)**, glucose production **(C)**, phosphoenolpyruvate carboxykinase (PEPCK) activity **(D)**, insulin receptor substrate (IRS)-1, and protein kinase B (Akt)1 phosphorylation **(E–G)** and glucose transporter 2 (GLUT2) expression **(H)**. Hierarchical cluster analysis and heat map [from the lowest (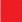 red) to the highest (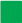 green) value for each parameter] **(I)** and an integrative diagram illustrating the effects of the bioactive compounds from coffee by-products on glucose metabolism **(J)**. The results are expressed as mean ± SD (*n* = 3). Bars with different letters significantly (*p* < 0.05) differ according to ANOVA and Tukey's multiple range test. NT, non-treated cells; PA, palmitic acid; CAF, caffeine; CGA, chlorogenic acid; CA, caffeic acid; PCA, protocatechuic acid; GA, gallic acid; KMP, kaempferol; FGF21, fibroblast growth factor 21.

NAFLD is also characterized by liver insulin resistance as well as glucose metabolism imbalance (73). We found a reduction in glucose absorption (45%) in NAFLD model HepG2 cells treated with PA (Figure 8A), suppressed by the bioactive compounds from coffee by-products and FGF21 (58–111%, *p* < 0.05). Additionally, PA-induced reduced GK activity was restored following the stimulation with coffee by-products bioactive compounds (55–122%, *p* < 0.05) (Figure 8B). Contrastingly, the *de novo* glucose synthesis was decreased by 20–39% (Figure 8C), which was most likely owing to the reduction (15–41%, *p* < 0.05) of PEPCK activity (Figure 8D), which is a critical catalyst in the gluconeogenic process. Concomitantly, the phosphorylation of IRS-1 and Akt1 and the expression of the GLUT2 were regulated (Figures 8E–H). CGA and PCA, among the other compounds, were the main regulators of glucose metabolism, enhancing the phosphorylation of IRS-1 by 1.9 and 2.1-fold, respectively (Figure 8F), and of Akt1 by 2.1 and 2.4-fold, respectively (Figure 8G). GLUT2 expression was augmented from 2.1 to 3.7-fold (*p* < 0.05) (Figure 8H). The hierarchical cluster analysis demonstrated that coffee husk bioactive compounds, CGA and PCA, can mimic FGF21 in regulating glucose metabolism and maintaining hepatocytes in basal conditions upon the PA stimulation (Figure 8I). GLUT2 is responsible for circulating glucose uptake into the liver (74). Absorbed glucose is phosphorylated by GK, resulting in glucose 6-phosphate, which lowers intracellular glucose concentrations while simultaneously increasing glucose uptake (Figure 8J). Accordingly, we noticed that the bioactive compounds identified in coffee by-products, particularly CGA and PCA, increased glucose absorption and GK activity. Similarly, FGF21 stimulates GLUT2-mediated glucose absorption and GK expression *in vivo* (75). Conversely, the expression/activation of major gluconeogenic proteins (PEPCK and glucose-6-phosphatase) and the presence of gluconeogenic substrates controls *de novo* glucose synthesis (Figure 8J) (74). CGA activates AMPK, leading to the suppression of hepatic glucose production by reducing G6Pase activity (76). PCA enhances PEPCK expression in dexamethasone-treated mice (77). FGF21 also has a role in the reduction of PEPCK and glucose synthesis (78). Steatosis in the liver leads to insulin resistance, favoring the development of NAFLD, generating a vicious cycle. As a result, insulin resistance impairs glucose absorption and insulin-mediated gluconeogenesis repression (74). Insulin and PI3K-Akt pathway regulation increase HepG2 insulin sensitivity, promoting glucose catabolic pathways, glucose absorption, and glycolysis while decreasing gluconeogenesis, among other anabolic pathways (79). Previous studies have shown that phytochemicals that increase the phosphorylation and activation of INSR, Akt, GSK3, and AMPK may also improve glucose absorption and decrease gluconeogenesis by decreasing PEPCK protein expression and activity (80). Similarly, bioactive compounds from coffee by-products may promote the ERK-activated mTOR/SK6 pathway, which is required to diminish gluconeogenesis and develop hyperglycemia and insulin resistance (81). Then, the bioactive compounds from coffee by-products, especially from the coffee husk, CGA, and PCA, may regulate glucose metabolism and avoid NAFLD development by preventing hyperglycemia and systemic insulin resistance.

### Coffee Husk Phenolics, Chlorogenic and Protocatechuic Acids and Kaempferol, Stimulated Hepatic Mitochondrial Bioenergetics and Energy Metabolism, Preventing NAFLD

**Figure 9:**
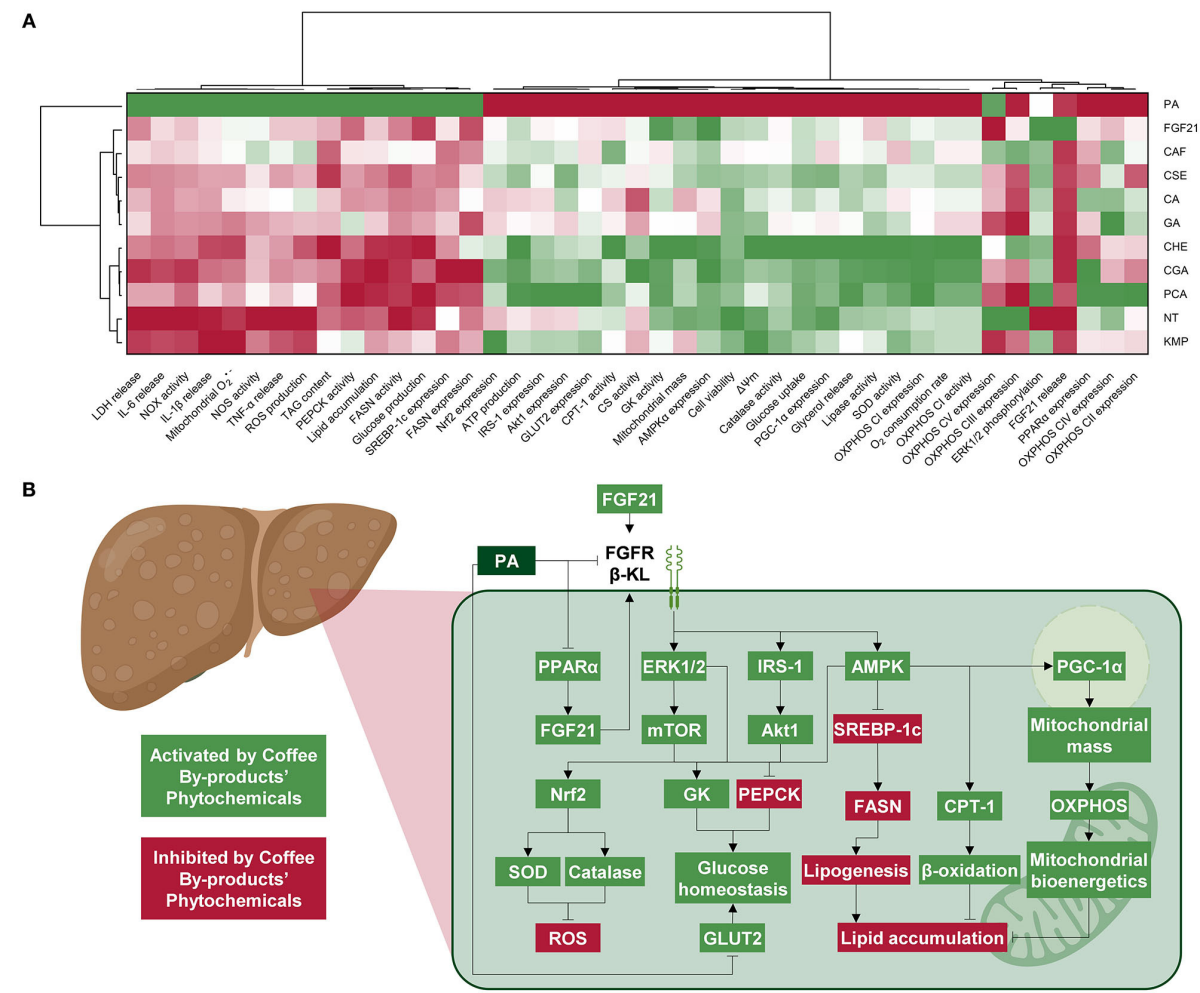
Integrative hierarchical cluster analysis and heat map (from the lowest (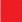 red) to the highest (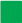 green) value for each parameter) unifying all parameters demonstrates that chlorogenic and protocatechuic acids are the bioactive compounds exhibiting the highest NAFLD-protecting effects **(A)**. Diagram illustrating the molecular mechanisms from the effects of the bioactive compounds in coffee by-products on hepatic FGF21 signaling, oxidative stress, mitochondrial bioenergetics, and energy metabolism **(B)**. NT, non-treated cells; PA, palmitic acid; CAF, caffeine; CGA, chlorogenic acid; CA, caffeic acid; PCA, protocatechuic acid; GA, gallic acid; KMP, kaempferol; FGF21, fibroblast growth factor 21.

The integrative estimation of the effects of bioactive compounds from coffee by-products in both the form of standard solutions and aqueous extracts using multivariate analysis demonstrated that at the concentrations tested, all treatments reverted PA induction of NAFLD (Figure 9A). The effects of CGA, PCA, and CHE were clustered together, suggesting their metabolism-promoting effects. In turn, KMP was clustered with the NT group, which indicated that this treatment preserved cell function without further stimulation. Finally, the other standard bioactive compounds, CSE and FGF21 were grouped. These treatments partially avoided PA adverse effects but also stimulated hepatic metabolism and mitochondrial bioenergetics. The molecular mechanisms of the bioactive compounds from the coffee silverskin and the coffee husk are depicted in Figure 9B. The activation of the FGF21 pathway and related signaling cascades lead to reduced oxidative stress and regulated mitochondrial bioenergetics and energy metabolism. It is worth noting that the concentration of the main compounds in coffee by-products is lower than the concentration of the standards evaluated (Supplementary Table 2). Effects from extracts may derive from a synergy between their components (82). Then, we could consider that using coffee by-products aqueous extracts may be a sustainable strategy for providing health-beneficial compounds. Industries usually prefer heat-assisted aqueous extractions where additional purification steps are not required; simple and quick extraction methods for revalorizing by-products minimize the adaptation of facilities, are cheaper, and save time and resources. It is also important to mention that the compounds found in coffee by-products could also be found in other food sources, and consequently, the effects described in this manuscript could also be inferred for mate tea or herbal teas (83, 84). Notwithstanding the bioactivity that bioactive compounds and aqueous extracts from coffee by-products have shown *in vitro*, their low bioavailability hinders their potential effectiveness in humans (85). These bioactive compounds are just moderately absorbed in the gastrointestinal tract and modified after digestion, microbiota fermentation, and liver metabolism (86). Hence, *in vivo* results may vary from those observed *in vitro*. To further comprehend the benefits of coffee by-products aqueous extracts and their compounds, additional studies should look at how digestion and metabolism in the gastrointestinal tract affect coffee silverskin and coffee husk bioactivity and the role of microbiota in that process. The bioefficacy and molecular mechanisms of action should also be validated using animal models. Both coffee silverskin and coffee husk have been proven as safe and sustainable ingredients; following acute (2 g kg^−1^ day^−1^, 1 day) and chronic (1 g kg^−1^ day^−1^, 90 days) administration in mice, no signs of toxicity were observed (87, 88). Nonetheless, further animal and clinical studies would be required to validate the beneficial properties reported *in vitro* and ascertain the gastrointestinal absorption and metabolism of bioactive compounds from coffee by-products.

## Conclusions

This research examined the effects of bioactive compounds and aqueous extracts from coffee by-products on FGF21 signaling activation, oxidative stress reduction, mitochondrial bioenergetics, and lipid and glucose metabolism regulation in HepG2 hepatocytes. Our findings show that the major bioactive compounds found in coffee by-products, namely chlorogenic and protocatechuic acids and kaempferol, may activate FGF21 signaling, reduce inflammation and oxidative stress, prevent mitochondrial dysfunction, and improve lipid and glucose homeostasis in HepG2 hepatocytes. In conclusion, our results evidenced that the bioactive compounds from coffee by-products could regulate hepatic mitochondrial bioenergetics and energy metabolism by activating FGF21 signaling. Moreover, we provide new insights into the molecular mechanisms of these bioactive compounds in coffee silverskin and coffee husk.

## Data Availability Statement

The original contributions presented in the study are included in the article/Supplementary Material, further inquiries can be directed to the corresponding author.

## Author Contributions

MR-H and EG: conceptualization. MR-H: formal analysis, investigation, data curation, and writing—original draft preparation. MR-H, YA, MM-C, and EG: writing—review and editing. MR-H and YA: visualization. YA, MM-C, and EG: supervision and funding acquisition. All authors have read and agreed to the published version of the manuscript.

## Funding

This research was funded by the USDA–NIFA–HATCH project (1014457), by the COCARDIOLAC project from the Spanish Ministry of Science and Innovation (RTI 2018-097504-B-I00), and the Excellence Line for University Teaching Staff within the Multiannual Agreement between the Community of Madrid and the UAM (2019–2023). MR-H. received funding from the Ministry of Universities for his predoctoral fellowship (FPU15/04238) and the support for an international research stay at the University of Illinois, Urbana-Champaign (EST18/0064).

## Conflict of Interest

The authors declare that the research was conducted in the absence of any commercial or financial relationships that could be construed as a potential conflict of interest.

## Publisher's Note

All claims expressed in this article are solely those of the authors and do not necessarily represent those of their affiliated organizations, or those of the publisher, the editors and the reviewers. Any product that may be evaluated in this article, or claim that may be made by its manufacturer, is not guaranteed or endorsed by the publisher.
