# Supplementary material for: Activating Effects of the Bioactive Compounds From Coffee By-Products on FGF21 Signaling Modulate Hepatic Mitochondrial Bioenergetics and Energy Metabolism in vitro
Source: Front Nutr. 2022 Mar 22;9:866233. doi: 10.3389/fnut.2022.866233 (PMC8981461; doi:10.3389/fnut.2022.866233)
Supplement: Supplementary file 1 [file Table_1.docx]

Supplementary Material

**Supplementary Table 1.** Bioactive compounds

| Protein name | Protein code | Biological function | Phosphorylation site | Effect of Phosphorylation |
| --- | --- | --- | --- | --- |
| *Insulin signaling* | | | | |
| Insulin-like growth factor1 receptor | IGF1R | Receptor tyrosine kinase which mediates actions of insulin-like growth factor 1 (IGF1). Binds IGF1 with high affinity and IGF2 and insulin (INS) with a lower affinity. Ligand binding activates the receptor kinase, leading to receptor autophosphorylation, and tyrosines phosphorylation of multiple substrates, that function as signaling adapter proteins including, the insulin-receptor substrates (IRS1/2), Shc and 14-3-3 proteins. | Y1165 | Induces activity |
| Insulin receptor | INSR | Receptor tyrosine kinase which mediates the pleiotropic actions of insulin. Binding of insulin leads to phosphorylation of several intracellular substrates. | Y1189 | Induces activity |
| Insulin receptor substrate 1 | IRS-1 | Plays a key role in transmitting signals from the insulin and insulin-like growth factor-1 (IGF-1) receptors to intracellular pathways PI3K-AKT and ERK-MAPK pathways. | S318 | Inhibits molecular association |
| SHC adaptor protein 1 | SHC-1 | Couples activated growth factor receptors to signaling pathways. Couples activated receptor tyrosine kinases to Ras via the recruitment of the GRB2/SOS complex, thus initiating the cytoplasmic proliferative Ras signaling cascade. | Y427 | Induces activity |
| Phosphatidylinositol 3,4,5-trisphosphate 5-phosphatase 1 | SHIP-1 | Phosphatidylinositol phosphatase that specifically hydrolyzes the 5-phosphate of phosphatidylinositol-3,4,5-trisphosphate (PtdIns(3,4,5)P3) to produce PtdIns(3,4)P2, thereby negatively regulating the PI3K pathway. | Y1020 | Induces activity |
| Tyrosine-protein phosphatase non-receptor type 11 | SHP-2 | SHP-2 association with Gab1 is critical for sustained ERK activation downstream of several growth factor receptors and cytokines. | T542 | Induces molecular association |
| *PI3K-Akt-PKB signaling* | | | | |
| Protein kinase B | Akt1 | Regulates cell survival and metabolism in many different signaling pathways. | S473 | Induces activity |
| B-cell lymphoma 2-associated death promoter | BAD | Initiates apoptosis. Displaces Bax from binding to Bcl-2 and Bcl-xL, resulting in cell death. | S112 | Inhibits molecular association |
| Glycogen synthase kinase-3 alpha | GSK3α | Constitutively active protein kinase that acts as a negative regulator in the hormonal control of glucose homeostasis. May also mediate the development of insulin resistance by regulating the activation of transcription factors. | S21 | Inhibits activity |
| Glycogen synthase kinase-3 beta | GSK3β | Constitutively active protein kinase that acts as a negative regulator in the hormonal control of glucose homeostasis. May also mediate the development of insulin resistance by regulating the activation of transcription factors. Probably regulates NF-κB at the transcriptional level and is required for the NF-κB-mediated anti-apoptotic response to TNF-α. | S9 | Inhibits activity |
| 3-phosphoinositide-dependent protein kinase 1 | PDK1 | Plays a central role in the transduction of signals from insulin by providing the activating phosphorylation to PKB/AKT1, thus propagating the signal to downstream targets controlling cell proliferation and survival, as well as glucose and amino acid uptake and storage. | S241 | Induces activity |
| Phosphatase and tensin homolog | PTEN | Dephosphorylates inositol phospholipids generated by the activation of PI3K. A major negative regulator of the PI3K/AKT signaling pathway. | S370 | Inhibits activity |
| *mTOR/S6K signaling* | | | | |
| Eukaryotic translation initiation factor 4E-binding protein 1 | 4E-BP1 | Binds to eIF4E, preventing its assembly into the EIF4F complex and inhibiting cap-dependent translation. Mediates the regulation of protein translation by hormones, growth factors signaling through the PI3K pathway. | T36 | Inhibits activity |
| Eukaryotic translation initiation factor 4E | EIF4E | Recognizes and binds the mRNA cap during an early step in the initiation of protein synthesis and facilitates ribosome binding by inducing the unwinding of the mRNAs secondary structures. | S209 | Inhibits molecular interaction |
| Mammalian target of rapamycin | mTOR | A downstream effector of PI3K activates AKT by phosphorylating a key activation site, activates p70S6K, and inactivates 4E-BP1, up-regulating protein synthesis. | T2448 | Induces activity |
| Ribosomal protein S6 kinase | p70S6K | Is required for cell growth and G1 cell cycle progression. Is phosphorylated and activated by mTOR in mitogenic pathways downstream of PI3K. | T421/S424 | Induces activity |
| Proline-rich AKT1 substrate 1 | PRAS40 | Subunit of mTORC1, which regulates cell growth and survival in response to nutrient and hormonal signals. mTORC1 is activated in response to growth factors or amino acids. Growth factor-stimulated mTORC1 activation involves a AKT1-mediated phosphorylation of TSC1-TSC2, which leads to the activation of the RHEB GTPase that potently activates the protein kinase activity of mTORC1. | T246 | Inhibits activity |
| 40S ribosomal protein S6 | rpS6 | Plays an important role in controlling cell growth and proliferation through the selective translation of particular classes of mRNA. | S235/236 | Induces activity |
| *FoxO signaling* | | | | |
| Forkhead box protein O3 | FOXO3 | Transcriptional activator which triggers apoptosis in the absence of survival factors, including neuronal cell death upon oxidative stress. In response to metabolic stress, translocates into the mitochondria where it promotes mtDNA transcription | S413 | Induces activity |
| Cyclin-dependent kinase inhibitor 1B | p27 | Important regulator of cell cycle progression. Involved in G1 arrest. | T198 | Inhibits molecular interaction |
| *AMPK signaling* | | | | |
| 5'-AMP-activated protein kinase catalytic subunit alpha | AMPKα | Acts as an energy sensor, playing a key role in regulating cellular energy metabolism. Activates energy-producing pathways and inhibits energy-consuming processes. | T172 | Induces activity |
| Liver kinase B1 | LKB1 | A tumor suppressor that helps control cell structure, polarity, apoptosis, and energy homeostasis. Activates AMPK and several related protein kinases. AMPK plays a predominant role as the master regulator of cellular energy homeostasis, controlling downstream effectors that regulate cell growth and apoptosis in response to cellular ATP concentrations. | S428 | Induces activity |
| Cellular tumor antigen p53 | p53 | Acts as a tumor suppressor in many tumor types; induces growth arrest or apoptosis depending on the physiological circumstances and cell type. Involved in cell cycle regulation as a trans-activator that acts to negatively regulate cell division by controlling a set of genes required for this process. | S15 | Induces activity |
| *MAPK signaling* | | | | |
| Mitogen-activated protein kinase 3 | ERK1/2 | Plays a critical role in the regulation of cell growth and differentiation. ERK1 and ERK2 play central roles in MAPK cascades mediating diverse biological functions such as cell growth, adhesion, survival, and differentiation through the regulation of transcription, translation, cytoskeletal rearrangements. | T202/Y204  Y185/187 | Induces activity |
| RAF proto-oncogene serine/threonine-protein kinase | Raf-1 | Serine/threonine-protein kinase that acts as a regulatory link between the membrane-associated Ras GTPases and the MAPK/ERK cascade, and this critical regulatory link functions as a switch determining cell fate decisions including proliferation, differentiation, apoptosis, survival and oncogenic transformation. | S301 | Inhibits activity |
| Ribosomal protein S6 kinase alpha-1 | RSK1 | Is involved in the mTOR nutrient-sensing pathway by directly phosphorylating TSC2 at 'Ser-1798', which potently inhibits TSC2 ability to suppress mTOR signaling, and mediates phosphorylation of RPTOR, which regulates mTORC1 activity and may promote rapamycin-sensitive signaling independently of the PI3K/AKT pathway | S380 | Induces protein degradation |
| Ribosomal protein S6 kinase alpha-3 | RSK2 | Is involved in the mTOR nutrient-sensing pathway by directly phosphorylating TSC2 at 'Ser-1798', which potently inhibits TSC2 ability to suppress mTOR signaling, and mediates phosphorylation of RPTOR, which regulates mTORC1 activity and may promote rapamycin-sensitive signaling independently of the PI3K/AKT pathway | S386 | Induces activity |

**Supplementary Table 2.** Bioactive compounds compositional profile of coffee silverskin (CSE) and coffee husk (CHE) aqueous extracts characterized by UPLC-ESI-MS/MS. Values are expressed as mean ± SD (*n* = 3).

| Compound | Concentration  (µg g^−1^ extract) | |  | Concentration (nmol L^−1^)  in 100 µg mL^−1^ treatments | | Chemical  structure |
| --- | --- | --- | --- | --- | --- | --- |
|  | CSE | CHE |  | CSE | CHE |  |
| ***Hydroxybenzoic acids*** |  |  |  |  |  |  |
| Gallic acid | 16.9 ± 1.2 | 87.0 ± 5.5 |  | 9.9 | 51.1 |  |
| Protocatechuic acid | 44.1 ± 3.4 | 488.4 ± 26.2 |  | 28.6 | 316.9 |  |
| 4-hydroxybenzoic acid | 3.4 ± 0.3 | 13.4 ± 1.3 |  | 2.5 | 9.7 |  |
| Vanillic acid | 29.6 ± 0.6 | 22.9 ± 9.6 |  | 17.6 | 13.6 |  |
| Salicylic acid | 2.3 ± 0.2 | 3.1 ± 0.1 |  | 1.7 | 2.2 |  |
| ***Hydroxycinnamic acids*** |  |  |  |  |  |  |
| Caffeic acid | 538.0 ± 54.3 | 57.9 ± 2.0 |  | 298.6 | 32.1 |  |
| Chlorogenic acid | 2791.7 ± 97.3 | 3456.8 ± 70.6 |  | 787.9 | 975.6 |  |
| *p*-coumaric acid | 0.9 ± 0.1 | 8.7 ± 0.2 |  | 0.5 | 5.3 |  |
| Ferulic acid | 3.8 ± 0.2 | N.D. |  | 2.0 | — |  |
| ***Mandelic acids*** |  |  |  |  |  |  |
| 3-hydroxymandelic acid | 4.4 ± 0.5 | N.D. |  | 2.6 | — |  |
| Mandelic acid | 5.1 ± 0.2 | N.D. |  | 3.4 | — |  |
| ***Phenylacetic acids*** |  |  |  |  |  |  |
| 3,4-dihydroxyphenylacetic acid | N.D. | 5.6 ± 2.0 |  | — | 3.3 |  |
| ***Flavan-3-ols: monomers*** |  |  |  |  |  |  |
| (+)-catechin | 10.2 ± 1.1 | 1.7 ± 0.2 |  | 3.5 | 0.6 |  |
| (‒)-epicatechin | N.D. | 18.0 ± 2.0 |  | 0.0 | 6.2 |  |
| ***Flavan-3-ols: dimers*** |  |  |  |  |  |  |
| Procyanidin B1 | N.D. | 22.3 ± 2.6 |  | — | 3.9 |  |
| Procyanidin B2 | N.D. | 11.6 ± 1.8 |  | — | 2.0 |  |
| ***Flavonols*** |  |  |  |  |  |  |
| Quercetin-3-*O*-galactoside | N.D. | 54.7 ± 0.5 |  | — | 11.8 |  |
| Quercetin-3-*O*-glucoside | N.D. | 57.4 ± 3.7 |  | — | 12.4 |  |
| Kaempferol-3-*O*-galactoside | N.D. | 122.6 ± 3.6 |  | — | 27.3 |  |
| ***Alkaloids*** |  |  |  |  |  |  |
| Caffeine | 19219.2 ± 37.6 | 9815.5 ± 15.4 |  | 9897.1 | 5054.6 |  |

N.D. Non-detected
